# Autophagy–Apoptosis Crosstalk in Cancer: Mechanisms, Signaling Pathways, and Therapeutic Targeting

**DOI:** 10.3390/cancers18101564

**Published:** 2026-05-12

**Authors:** Dia Kakkar, Saloni Saxena, Utkarshita Dhawan, Naman Dosi, Charvi Khanna, Souren Paul

**Affiliations:** Cell and Molecular Biology Lab, TERI School of Advanced Studies, Plot No. 10, Sankar Rd, Vasant Kunj Institutional Area, New Delhi 110070, India; dia.kakkar@terisas.ac.in (D.K.); saloni.saxena@terisas.ac.in (S.S.); utkarshita.dhawan@terisas.ac.in (U.D.); naman.dosi@terisas.ac.in (N.D.)

**Keywords:** autophagy, apoptosis, crosstalk, kinase, transcription factors, caspases, MAPK, UPR, redox signaling, HSPs, calcium signaling

## Abstract

Autophagy and apoptosis are two evolutionarily conserved catabolic processes that play important roles in maintaining cellular homeostasis and in determining cell fate when cells are exposed to various stresses in vivo. The interaction between autophagy and apoptosis has been studied extensively in cancer research, and it has been shown to affect cancer initiation and tumor formation, disease progression, therapeutic resistance, and overall survival. Autophagy typically functions as a cytoprotective mechanism in cancer cells subjected to metabolic, hypoxic, or therapeutic stress, whereas apoptosis primarily functions as an intrinsic programmed cell death pathway. While apoptosis and autophagy function as distinct pathways, there is significant molecular crosstalk, allowing cells to modulate their behavior from survival to death depending on the severity and duration of exposure to a given stressor and the cellular environment. This review examines the molecular landscape of the autophagy–apoptosis interplay in cancers, with special attention paid to the major signaling pathways involved and their biological outcomes in oncology. We examine the molecular mechanisms and signal transduction pathways involved in the crosstalk between autophagy and apoptosis in cancer. In particular, we focus on several key proteins that regulate this crosstalk, including kinases, caspases, heat shock proteins and transcription factors. Furthermore, we describe the major signal transduction pathways that regulate this crosstalk, including the PI3K/Akt/mTOR, MAPK, unfolded protein response, oxidative stress, and calcium signaling pathways. Additionally, we discussed how dysregulation of these pathways contributes to cancer progression and treatment resistance. Finally, we summarized the use of currently available therapeutic agents targeting the crosstalk between autophagy and apoptosis, including FDA-approved drugs and natural products, with the potential to enhance the effectiveness of anticancer treatments. A better understanding of this complex process will allow the development of new, precision-based, combination cancer therapies.

## Simple Summary

Autophagy and apoptosis are two critical processes that determine whether a cell lives or dies in response to cellular stress. Both play significant roles in how cancer forms, grows, responds to treatments, and affects patient survival. Although autophagy and apoptosis play different roles within a cell, they share common pathways and molecular signals that enable them to communicate. As a result, cells exposed to stress can switch from survival to death based on the severity of stress. This article identifies the primary molecules and signal transduction pathways that regulate the connection between autophagy and apoptosis in cancer cells. These include regulatory proteins and pathways such as PI3K/Akt/mTOR, MAPK, redox balance, and calcium signaling. The article also reviews how defects in these networks facilitate cancer progression and resistance to therapy. It critically assesses existing drugs and natural compounds that can disrupt this interaction to enhance cancer treatment outcomes.

## 1. Introduction

Autophagy and apoptosis are two catabolic pathways that have been shown to be involved in cellular homeostasis and in determining cell fate under stressful conditions [1]. The intricate balance between these cellular processes is a field of intense study in cancer research due to its implications for tumorigenesis and therapeutic options [2,3]. These processes frequently occur together in the tumor microenvironment; therefore, understanding their interactions is important for predicting cellular outcomes and developing effective cancer therapies [4]. Autophagy, a form of lysosome-dependent programmed cell death, and apoptosis, another form of programmed cell death, are critical processes that help maintain homeostasis at the organismal level [3,5,6,7]. Autophagy generally protects cells against intermediate levels of stress, but failing to do so at higher levels leads to apoptosis [1]. When autophagy and apoptosis function together, they can crosstalk via shared regulatory proteins and signaling pathways. This crosstalk enables cells to transition from a survival to a death mode in response to changes in cellular stress [5,8]. However, under certain conditions, components of the autophagy pathway can also induce apoptosis to minimize tissue damage [9]. The molecular events that govern whether autophagy leads to apoptosis include the interaction between Beclin-1 and the anti-apoptotic B-cell lymphoma 2 (Bcl-2) and the regulation of downstream signaling cascades by protein kinases and transcription factors [5,10].

Previous studies have shown that many chemotherapeutic drugs and radiation therapy create metabolic stress in cancer cells. When combined with autophagy inhibition, it provides a rationale for modulating autophagy in anti-cancer treatment approaches [11,12]. In addition to being controlled by numerous regulators, autophagy and apoptosis are simultaneously regulated by multiple regulators, suggesting that targeting both pathways may be the most efficient strategy for limiting tumor progression [5,13]. Understanding the complex relationship between these two processes may also guide clinicians in selecting appropriate treatment modalities and predicting patient response [14,15]. We described the molecular mechanisms linking autophagy and apoptosis, including how protein kinases and transcription factors regulate signaling pathways that determine cellular fate. This review also discusses the roles of other key proteins, such as caspases and heat shock proteins (HSPs), in autophagy–apoptosis crosstalk. This manuscript also aims to discuss significant signaling mechanisms that influence autophagy–apoptosis crosstalk and, lastly, discuss current therapeutic opportunities targeting all these proteins and pathways.

## 2. Proteins Involved in Autophagy–Apoptosis Crosstalk

A comprehensive understanding of the molecular mechanisms and protein–protein interactions that regulate crosstalk between autophagy and apoptosis in cancer is essential for developing targeted therapies [16]. Several protein families have been identified as potential regulators of both autophagy and apoptosis, including kinases, transcription factors, caspases, mitogen-activated protein (MAP) kinase proteins, and HSPs [16,17].

### 2.1. Kinases

#### 2.1.1. Death-Associated Protein Kinase (DAPK)

**Figure 1:**
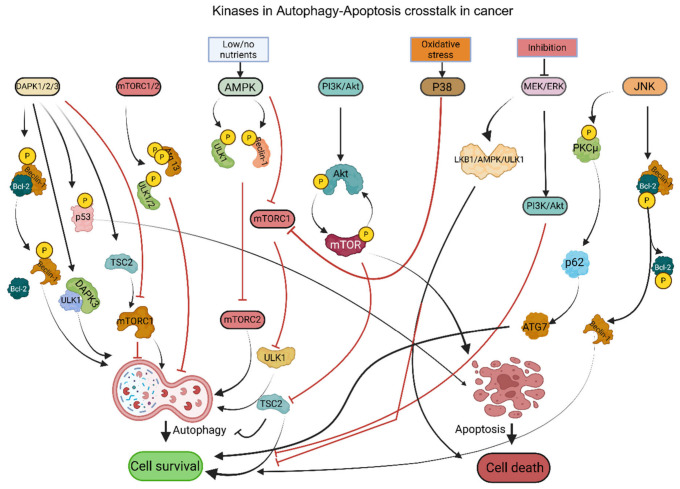
Role of kinases in autophagy–apoptosis crosstalk in cancer. Figure 1 illustrates the specific roles of key kinases and their crosstalk in regulating autophagy and apoptosis. The black arrow indicates activation, and the red line indicates inhibition.

DAPK is a tumor suppressor kinase that regulates autophagy and apoptosis in a context-dependent manner by phosphorylating proteins involved in stress signaling, cytoskeletal dynamics, and cell death pathways [18,19]. Phosphorylation of Beclin-1 by DAPK at Thr119 induces autophagy through disruption of the interaction between Beclin-1 and anti-apoptotic proteins such as Bcl-XL or Bcl-2, thereby freeing Beclin-1 to participate in the class III PI3K/Vps34 complex required for autophagosome initiation [20]. DAPK1 induces apoptosis by phosphorylating p53 at Ser20, activating ARF, and facilitating calcium ion (Ca^2+^) influx through GluN2B, thereby enhancing pro-apoptotic signaling, stress checkpoint activation, and calcium-dependent cell death pathways [21] (Figure 1; Table 1). In fact, DAPK can also facilitate Mechanistic target of rapamycin complex 1 (mTORC1) activation by phosphorylating Tuberous sclerosis complex 2 (TSC2) and thus typically suppresses autophagy under certain cellular conditions, highlighting its dual regulatory role in autophagy depending on upstream stress cues and isoform-specific signaling [9,22] (Figure 1; Table 1). DAPK2 is unique in the regulation of autophagy induction through the negative modulation of mTORC1 activity, whereas DAPK1 is involved in the nucleation of autophagosomes via the Vps34 complex (Figure 1; Table 1), suggesting functional divergence among DAPK family members in controlling early autophagy signaling [23]. Additionally, DAPK1’s cytoplasmic localization allows for its phosphorylation of myosin light chain and subsequent actomyosin contractility and membrane blebbing during apoptosis, both of which are characteristic morphological features of apoptotic cell dismantling [24]. DAPK3 is involved in autophagosome formation through interactions with the autophagy-related 1 protein kinase and may contribute to autophagy initiation by regulating cytoskeletal and membrane remodeling events [25] (Figure 1; Table 1). DAPK is a key mediator of apoptosis activated by many stimuli, including interferon γ, transforming growth factor β, and ceramides, placing it at the intersection of cytokine signaling, stress adaptation, and programmed cell death regulation [19].

#### 2.1.2. Mechanistic Target of Rapamycin (mTOR)

mTOR is a central serine/threonine kinase that forms two complexes, mTORC1 and mTOR complex 2 (mTORC2), each defined by distinct scaffold proteins and downstream signaling functions [26]. mTOR exists in two structurally and functionally distinct complexes, mTORC1 and mTORC2, which exert differential effects on autophagy and cellular signaling [27]. mTORC1 primarily functions as a nutrient- and energy-sensing complex that regulates protein synthesis, lipid metabolism, and cell growth, while acting as a central negative regulator of autophagy [27,28]. Mechanistically, mTORC1 regulates anabolic processes and inhibits catabolic processes, such as autophagy, by phosphorylating downstream targets, including Unc-51-like autophagy activating kinase 1/2 (ULK1/2) and Atg13 (Figure 1; Table 1), thereby preventing activation of the Unc-51-like autophagy activating kinase 1 (ULK1) initiation complex and suppressing autophagosome formation under nutrient-rich conditions [29]. In contrast, mTORC2 primarily regulates cytoskeletal organization and cell survival by activating kinases such as Akt, SGK, and PKC, which support cell growth, metabolism, and survival signaling [30]. Unlike mTORC1, the role of mTORC2 in autophagy is more complex and context-dependent, involving indirect regulation through AKT–FOXO signaling and transcriptional control of autophagy-related genes [31,32]. Thus, while mTORC1 directly inhibits autophagy initiation, mTORC2 modulates autophagy more indirectly and can either suppress or fine-tune autophagic responses depending on cellular context [31]. Importantly, crosstalk between the two complexes further refines cellular outcomes, as mTORC1 activity can negatively regulate mTORC2 signaling via feedback mechanisms, highlighting their coordinated yet distinct roles [33]. Under stress conditions such as amino acid deprivation or elevated intracellular calcium, DAPK (particularly DAPK2) promotes autophagy by inhibiting mTORC1 activity through phosphorylation of its regulatory components, thereby facilitating autophagic induction [33,34] (Figure 1; Table 1). Consistently, recent studies demonstrate that DAPK2-mediated suppression of mTORC1 enhances autophagic flux and contributes to stress adaptation and cellular remodeling [34]. Conversely, in specific cellular contexts, including during cancer progression, DAPK signaling can promote cell survival or proliferation, indirectly limiting autophagy or shifting its functional outcome toward tumor-promoting processes. This apparent contradiction reflects the broader dual nature of autophagy itself, which can function either as a tumor-suppressive mechanism or as a pro-survival pathway depending on the cellular and microenvironmental context [35]. Therefore, the role of DAPK in autophagy should be interpreted in a context-dependent manner, where it promotes autophagy under stress via mTORC1 inhibition, but may also contribute to survival pathways that attenuate autophagic cell death in certain disease states [34,35].

#### 2.1.3. AMP-Activated Protein Kinase (AMPK)

AMPK functions as a major energy sensor and counteracts the mTORC1 pathway by increasing the AMP/ATP and ADP/ATP ratios during metabolic stress. When cellular energy levels are low, AMPK initiates autophagy by directly phosphorylating and activating ULK1 and Beclin-1, and by inhibiting mTORC1 (Figure 1; Table 1), thereby coordinating energy conservation with activation of the autophagy initiation machinery [36,37]. AMPK can influence autophagy by inhibiting mTOR via phosphorylation of TSC2 and Raptor, and by promoting activation of the ULK1 complex, which is required for phagophore formation and early autophagosome biogenesis [38] (Figure 1; Table 1). Conversely, under nutrient deprivation, AMPK is activated to inhibit mTORC1, activate ULK1, and induce autophagy (Figure 1; Table 1), therefore, enabling cells to recycle intracellular components to restore metabolic homeostasis [39]. Moreover, AMPK induces intrinsic cell death by increasing the Bax/Bcl-2 ratio, activating caspase-9 and subsequently caspase-3, and promoting PARP cleavage. AMPK also suppresses cell-survival pathways such as Akt/mTOR, leading to programmed cell death [40,41].

#### 2.1.4. Phosphoinositide 3-Kinase (PI3K)/Akt

This central signaling cascade has been demonstrated to consolidate extracellular and intracellular signals to control cell growth, proliferation, and survival by coupling growth factor receptor activation to downstream metabolic and biosynthetic programs [42]. Dysregulation of this pathway is common in cancer and promotes tumor growth while inhibiting apoptosis, making it a potential therapeutic target via sustained activation of survival, proliferation, and nutrient-sensing signals [43]. Activation of PI3K leads to Akt activation through the generation of PIP3 at the plasma membrane and recruitment of PDK1 and Akt, and Akt, in turn, promotes mTOR phosphorylation, which promotes cell growth and inhibits autophagy primarily through activation of mTORC1-dependent anabolic signaling [42,44] (Figure 1; Table 1). The direct phosphorylation of Akt by mTORC2 amplifies pro-survival signaling by enhancing Akt kinase activity and reinforcing downstream inhibition of apoptotic and autophagic pathways [45] (Figure 1; Table 1). Growth factors stimulate the PI3K/Akt pathway to inhibit autophagy by activating mTORC1 via inhibition of the Tuberous sclerosis complex 1/2 (TSC1/2) complex through relieving the suppression of Rheb and enabling mTORC1 activation [46,47] (Figure 1; Table 1).

#### 2.1.5. p38 Mitogen-Activated Protein Kinase (p38 MAPK)

p38 MAPK is a key member of stress-activated pathways and modulates autophagy based upon cellular stressors such as oxidative stress, inflammatory cytokines, DNA damage, etc. [48]. Depending upon the type of cellular stress and cellular context, p38 MAPK may either induce or suppress autophagy, reflecting the context-dependent balance between adaptive stress signaling and pro-death responses [49]. p38 MAPK may induce autophagy through the inhibition of mTORC1 and activation of ULK1, as a result of cellular oxidative stress (Figure 1; Table 1), thereby promoting autophagy initiation under conditions that threaten cellular homeostasis [50]. p38 MAPK also may induce apoptosis through enhancing p53 transcriptional activity and concomitant Bax-mediated mitochondrial cell death. P38 MAPK also activates caspase-3 cleavage to induce cell death [51,52].

#### 2.1.6. Mitogen-Activated Protein Kinase (MEK)/Extracellular Signal-Regulated Kinase (ERK)

The MEK/ERK pathway is another regulator of autophagy; activation of the MEK/ERK pathway may promote starvation-induced autophagy, and reactive oxygen species (ROS)-dependent ERK activation may enhance autophagy and induce cell death by modulating stress-responsive signaling networks and autophagy-related regulatory proteins [53,54]. Inhibition of ERK1/2 may induce autophagy through the LKB1/AMPK/ULK1 pathway, particularly in pancreatic ductal adenocarcinoma cells [55,56] (Figure 1; Table 1), suggesting that ERK1/2 can function as a context-dependent suppressor of autophagy in certain tumor settings.

#### 2.1.7. c-Jun N-Terminal Kinase (JNK)

JNK is another critical component of the MAPK superfamily that promotes or suppresses autophagy, depending on the type and severity of cellular stress, including oxidative stress, ER stress, inflammatory signaling, and DNA damage [57,58]. Phosphorylation of Bcl-2 by JNK releases Beclin-1 from the Bcl-2-Beclin-1 complex, resulting in the initiation of autophagosomes formation by enabling Beclin-1 to associate with the class III PI3K/Vps34 complex required for autophagosome nucleation [59] (Figure 1; Table 1). Moreover, JNK facilitates autophagy by phosphorylating PKCμ, leading to the subsequent recruitment of Sequestosome 1 (p62) and promotion of selective autophagic signaling. JNK also facilitates autophagy by activating ATG7, which supports LC3 conjugation and autophagosome elongation [60,61] (Figure 1; Table 1). Activation of JNK is associated with the generation of reactive oxygen species (ROS) mediated cell death, particularly when sustained JNK signaling amplifies oxidative damage and shifts the balance from adaptive autophagy toward apoptosis or other forms of stress-induced cell death [62].

### 2.2. Caspases

**Figure 2:**
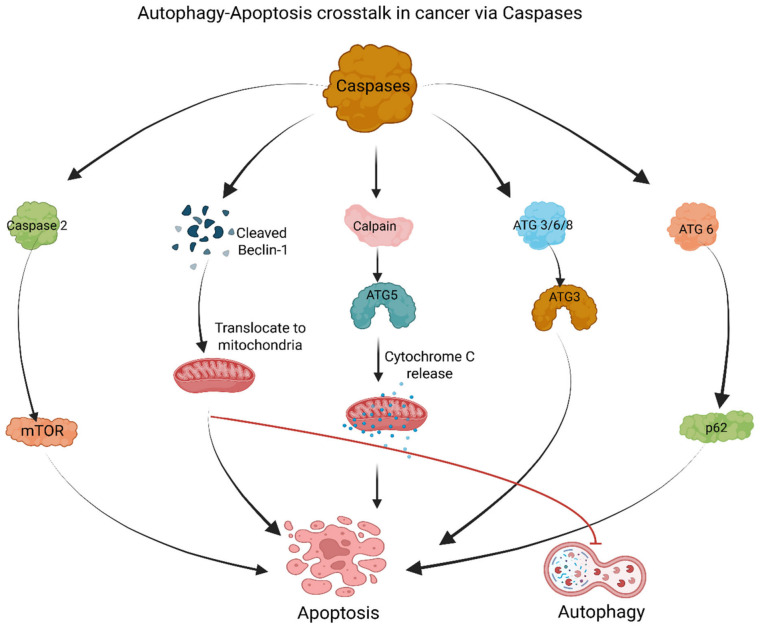
Role of caspases in autophagy–apoptosis crosstalk in cancer. Figure 2 illustrates the interconnected pathways through which caspases regulate the induction of autophagy or apoptosis. The black arrow indicates activation, and the red line indicates inhibition.

Caspases were previously considered the primary “executioner” molecules involved in apoptosis; however, they can also influence cell fate by modifying autophagy-related proteins, and vice versa [10]. For example, cleavage of Beclin-1 by caspases yields a C-terminal fragment that translocate to the mitochondrial outer membrane, where it can promote mitochondrial outer membrane permeabilization (MOMP) and enhance cytochrome c release, and enhances cell apoptosis [63,64] (Figure 2; Table 2). Since Beclin-1 initiates autophagy, its cleavage during apoptosis inhibits autophagy by disrupting the Beclin-1–Vps34/class III PI3K complex required for autophagosome nucleation [64]. The report suggests that regulatory proteins of autophagic pathways can directly influence caspase activity [65]. Autophagy-related proteins like ATG5, ATG12, and ATG16L1 inhibit pro-caspase-8 processing and, subsequently, apoptosis, likely by sequestering caspase-8 at autophagosomal membranes and preventing its full activation [66]. However, cleavage of ATG5 by calpain results in mitochondrial localization, leading to cytochrome c release and concomitant apoptosis (Figure 2; Table 2), in part through interaction with anti-apoptotic Bcl-2 family proteins such as Bcl-XL [10,67]. Similarly, caspases can cleave other autophagy-related proteins, such as Atg3, Atg4D, and Atg7, with effects that vary depending on the type of cleavage product and cellular context; for example, cleavage of Atg3 and Atg7 disrupt LC3 conjugation systems, whereas cleavage of Atg4D may generate a fragment with pro-apoptotic mitochondrial activity [68]. Therefore, these interactions together represent a finely tuned system in which the timing and degree of cleavage events determine whether cells undergo autophagy-induced survival or apoptosis-induced death [69,70].

While activated caspases can inhibit autophagy by degrading autophagy-related proteins and shift the cellular response towards apoptosis, autophagy can regulate apoptosis by modulating caspase levels and activity, e.g., by clearing caspase-8 via autophagy (Figure 2; Table 2) and thereby limiting death receptor-mediated apoptotic signaling [10]. Furthermore, anti-apoptotic proteins such as FLIP can modulate crosstalk between autophagy and apoptosis by competing with ATG3 for binding to the identical ligands, thereby blocking LC3 lipidation and suppressing autophagosome membrane expansion [9] (Figure 2; Table 2). It has also been demonstrated that caspases-6, -8, and -3 can cleave ATG3, thereby disrupting autophagosome formation and autophagy, whereas caspase-6 can also cleave p62, which also affects autophagy by impairing cargo recognition and selective autophagic degradation [61,67] (Figure 2; Table 2). In addition, caspase involvement in non-apoptotic cell death processes, such as necroptosis and pyroptosis, indicates that caspases play critical roles in maintaining cellular homeostasis and in disease development [71]. Dysfunction of caspases has been identified in various diseases, including cancer, neurodegenerative disorders, and inflammatory diseases; therefore, caspases represent promising therapeutic targets [72,73].

Among the initiator caspases, caspase-2 is evolutionarily conserved and plays two distinct roles in apoptosis and autophagy [74]. Specifically, under conditions of cellular stress, including DNA damage and heat shock, caspase-2 promotes apoptosis through stress-responsive signaling complexes such as the PIDDosome and downstream mitochondrial apoptotic pathways, whereas under basal conditions, caspase-2 suppresses autophagy [74,75] (Figure 2; Table 2). However, loss of caspase-2 was shown to result in increased autophagy, especially under oxidative stress, through multiple pathways including those initiated by AMPK, MAPK1/3, down-regulated Mechanistic target of rapamycin (mTOR), or inactivated MAPK14 (Figure 2; Table 2), all of which converge on activation of the ULK1 complex and induction of autophagic flux [76]. Additionally, caspase-10, a close homolog of caspase-8, has been found to regulate autophagy by downregulating BCL2-associated transcription factor 1 (BCLAF1), thereby reducing excessive autophagy in myeloma cells, suggesting a non-canonical role for initiator caspases in balancing survival and death pathways [10,77].

### 2.3. Heat Shock Proteins

**Figure 3:**
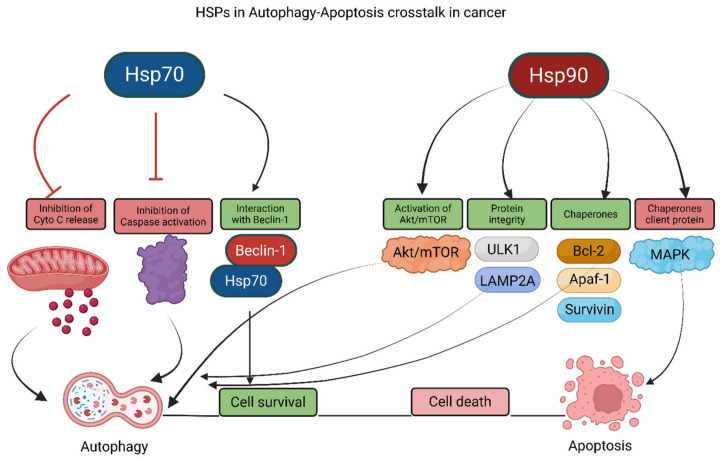
Role of heat shock proteins in autophagy–apoptosis crosstalk. Figure 3 illustrates how heat shock proteins (HSPs), particularly HSP70 and HSP90, mediate cell fate decisions through autophagy or apoptosis. The black arrow indicates activation, and the red line indicates inhibition.

HSPs are important molecular chaperones that serve as cellular stress responders, determining cellular fates towards autophagy and apoptosis [7,78]. Reports suggest that elevated HSP expression is generally associated with malignancy, therapy resistance, and radiation resistance, largely due to their ability to stabilize oncogenic signaling proteins and suppress proteotoxic stress-induced cell death [79,80]. Therefore, HSPs serve as both prognostic and diagnostic markers in cancer [81]. HSP70 and HSP90 have been studied extensively for their roles in balancing autophagy and apoptosis in cells [82]. HSP70 expression hinders cell death by limiting cytochrome c release from mitochondria and caspase activation, partly by stabilizing mitochondrial membrane integrity and inhibiting apoptosome assembly [83]. Moreover, HSP70 can regulate autophagy by directly interacting with Beclin-1 (Figure 3) and may facilitate proper folding or stabilization of components involved in autophagosome initiation [84].

Interestingly, the effect of HSP90 on autophagy–apoptosis crosstalk depends on the types of client proteins it is chaperoning (Figure 3), as HSP90 supports the conformational maturation and stability of numerous kinases, transcription factors, and apoptosis regulators [85,86,87]. HSP90 can also inhibit protein aggregation, reduce cellular stress and potentially prevent the activation of pro-apoptotic and exaggerated autophagy signals by maintaining proteostasis under stress conditions [88] (Figure 3). The HSP90/AKT/mTOR axis is the central pathway that balances autophagy–apoptosis crosstalk via HSP90 [89]. In addition, HSP90 can interfere with the Akt, Raf/Ras, and MAP kinase pathways by stabilizing their active conformations and protecting them from ubiquitin-proteasome-mediated degradation [90] (Figure 3).

Moreover, unlike direct activation of Akt by HSP90, activation of mTOR by HSP90 is indirect and occurs primarily through stabilization of upstream regulators such as Akt and other growth-promoting signaling intermediates [91]. Activation of Akt by HSP90 is associated with increased autophagic flux and cell survival, although in many contexts Akt also suppresses canonical autophagy through mTOR complex 1 (mTORC1) activation, highlighting the context-dependent nature of HSP90 signaling [92] (Figure 3). This dual role where Akt suppresses the initiation of autophagosome formation while simultaneously promoting the fusion of autophagosomes with lysosomes creates a complex biochemical landscape with profound implications for oncology and neurodegeneration. Furthermore, because the functional stability of Akt is intrinsically dependent on the HSP90 chaperone complex, pharmacological targeting of HSP90 has emerged as a high-priority strategy to disrupt this survival signaling hub. This report provides an exhaustive examination of the Akt-autophagy paradox, the molecular mechanics of the HSP90-Akt interaction, and the clinical trajectory of HSP90 inhibitors from early ansamycin natural products to the recent regulatory approval of pimitespib [93,94,95,96]. A list of HSP-90-targeted therapeutics is presented in Table 3 [97,98,99,100,101,102,103,104,105,106,107].

HSP90 also helps maintain Akt’s phosphorylation state by inhibiting its dephosphorylation and by shielding Akt from phosphatases such as Protein Phosphatase 2A (PP2A) and PH domain and Leucine-rich repeat Protein Phosphatase (PHLPP) [108]. HSP90 also plays a vital role in protecting protein integrity of ULK1, a primary autophagy activator, as well as Lysosome-associated membrane protein 2A (LAMP2A), an essential protein for chaperone-mediated autophagy (Figure 3), thereby sustaining both macroautophagy initiation and selective lysosomal substrate uptake [109].

Furthermore, inhibition of HSP90 results in Akt degradation and induces autophagic cell death by activating the Akt/mTOR pathway (Figure 3); more precisely, HSP90 inhibition destabilizes Akt, suppresses mTOR signaling, relieves inhibition on ULK1, and ultimately promotes autophagy, which under certain stress conditions can become excessive and cytotoxic [110,111]. In addition, HSP90 chaperones other apoptosis regulators, including BCL-2, Apaf-1, and Survivin (Figure 3), thereby influencing both mitochondrial apoptosis and caspase-dependent survival signaling [112]. The report also suggests that mitochondrial isoforms of HSP90 play essential roles in the development of resistance to cell death by regulating metabolism and mitochondrial function, including the maintenance of oxidative phosphorylation, mitochondrial protein folding, and the suppression of mitochondrial permeability transition [113,114]. These findings suggest that HSP90 can be targeted to modulate autophagy–apoptosis crosstalk and may represent a potential therapeutic target.

### 2.4. Transcription Factor

Transcription factors have been identified as key mediators of crosstalk between autophagy and apoptosis [115]. Therefore, a comprehensive understanding of the regulatory role of transcription factors on the rationale between autophagy and apoptosis may provide new ways to treat cancer [116,117]. Several transcription factors have been shown to influence crosstalk between autophagy and apoptosis, but this review focuses on a few key ones.

#### 2.4.1. C/EBP Homologous Protein (CHOP)

CHOP, also known as Growth arrest and DNA damage-inducible protein 153 (GADD153), is a bZIP transcription factor that induces apoptosis and autophagy in response to endoplasmic reticulum (ER) stress and is primarily regulated downstream of the PERK–eIF2α–ATF4 branch of the unfolded protein response (UPR) [118,119]. Although normally expressed at low levels, CHOP is induced to high levels under pathological conditions of ER stress due to sustained translational attenuation and selective transcriptional activation mediated by ATF4 and other UPR-associated factors [120].

**Figure 4:**
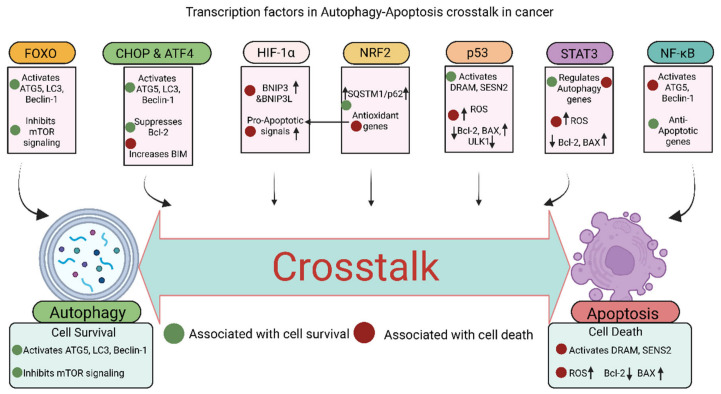
Roles of different transcription factors in autophagy–apoptosis crosstalk in cancer. Figure 4 illustrates how various transcription factors regulate the induction of autophagy or apoptosis and their association with cell survival or cell death. Up arrow indicates upregulation; down arrow indicates downregulation.

The induction of CHOP results in the promotion of mitochondria-dependent apoptosis by suppression of the anti-apoptotic Bcl-2 family members through transcriptional repression mechanisms; increase in BIM via direct transcriptional upregulation of pro-apoptotic BCL2 homology 3 (BH3)-only genes; increase in oxidative stress due to increased expression of ERO1α, which enhances disulfide bond formation and generates reactive oxygen species (ROS); disruption of Ca^2+^ homeostasis by promoting ER calcium release and mitochondrial calcium overload; and inhibition of AKT signaling through TRB3 (Figure 4; Table 4), where TRB3 acts as a pseudokinase that binds and inhibits Akt phosphorylation [118,121,122]. CHOP also promotes apoptosis by inducing pro-apoptotic genes such as BIM, PUMA, and NOXA under severe ER stress conditions [123].

Additionally, the induction of CHOP promotes autophagy by activating ATGs such as ATG5 and ATG7 through transcriptional regulation and reducing anti-autophagic regulators, including suppression of Bcl-2-mediated inhibition of Beclin-1; thus, CHOP acts as a molecular switch that determines whether cells will survive or die during times of stress (Figure 4; Table 4), depending on the intensity and duration of ER stress and the balance between adaptive UPR signaling and pro-death pathways [124].

#### 2.4.2. Activating of Transcription Factor 4 (ATF4)

ATF4, a key modulator of autophagy under conditions of endoplasmic reticulum (ER) stress, is an essential downstream effector of the PERK signaling pathway and is selectively translated following PERK-mediated phosphorylation of Eukaryotic initiation factor 2 alpha (eIF2α) during the UPR [125,126]. ATF4 activates autophagy by regulating genes involved in autophagy, such as MAP1LC3, ATG12, and Beclin1, typically in association with CHOP through transcriptional activation of stress-responsive promoters that enhance autophagosome formation and autophagic flux [124,127] (Figure 4; Table 4).

ATF4 also directly interacts with the CRE element in the LC3B promoter to regulate LC3B expression and autophagy under the most stressful conditions, including hypoxia (Figure 4; Table 4), thereby linking integrated stress signaling to increased LC3B synthesis and autophagosome membrane expansion [59,128]. ATF4 serves as a link between the ER stress response and the autophagy-lysosome system, thereby regulating the equilibrium between autophagy and apoptosis by coordinating adaptive metabolic reprogramming, redox balance, amino acid homeostasis, and stress-induced survival or death pathways, and may be a new therapeutic target for cancer treatment [17]. ATF4 contributes to apoptosis primarily by inducing CHOP (DDIT3), which subsequently activates pro-apoptotic signaling under prolonged ER stress [123].

#### 2.4.3. Forkhead Box O (FOXO)

The FOXO family of transcription factors, especially FOXO1, is essential to both apoptosis and autophagy regulation in response to ER stress, functioning as stress-responsive transcriptional regulators downstream of signaling pathways such as PI3K/Akt and AMPK [129,130]. The unspliced form of the protein X-box binding protein 1 (XBP1u) promotes proteasome-mediated degradation of FOXO1, thereby limiting autophagy; conversely, the absence of X-box binding protein 1 (XBP1) allows sustained FOXO1-driven autophagy (Figure 4; Table 4), likely by preserving FOXO1 protein stability and prolonging its transcriptional activity in the nucleus [131].

ER stress activates FOXO1 and induces the expression of autophagy-related genes ATG5, LC3, and Beclin-1 through direct binding of FOXO1 to promoter regions of autophagy-associated genes and enhancement of autophagic flux [132] (Figure 4; Table 4). Conversely, the spliced version of XBP1 (XBP1s) increases autophagy by elevating Beclin-1 levels and promoting LC3-II formation, suggesting that distinct XBP1 isoforms differentially regulated autophagy depending on the context and duration of ER stress [59,124]. Under oxidative stress conditions, FOXO1 can localize to either promote autophagy at lower stress levels as part of an adaptive survival response involving antioxidant defense and cellular recycling or to promote apoptosis at higher stress levels by inducing pro-apoptotic genes and shifting the balance from stress adaptation to irreversible cell death [133] (Figure 4; Table 4). FOXO transcription factors regulate apoptosis by inducing pro-apoptotic genes such as BIM and coordinating stress-responsive pathways under conditions of nutrient deprivation and oxidative stress [134,135,136].

#### 2.4.4. Hypoxia-Inducible Factor-1α (HIF-1α)

HIF-1α is the key transcription factor linking hypoxia to autophagy and apoptosis by inducing genes involved in angiogenesis, metabolism, and stress responses under low-oxygen conditions, thereby affecting cell fate [137,138]. Under normoxic conditions, HIF-1α is hydroxylated by prolyl hydroxylases and targeted for VHL-mediated proteasomal degradation, whereas hypoxia stabilizes HIF-1α, allowing its nuclear accumulation and transcriptional activity [139].

The role of HIF-1α in promoting autophagy is mediated by increased expression of BCL2 interacting protein 3 (BNIP3) and BCL2 interacting protein 3-like (BNIP3L); this allows the cell to survive hypoxic stress by disrupting the inhibitory interaction between Beclin-1 and anti-apoptotic Bcl-2/Bcl-XL proteins and by facilitating mitophagy of damaged mitochondria [140,141] (Figure 4; Table 4). However, in addition to activating autophagy, HIF-1α may also induce pro-apoptotic programs under extreme or prolonged hypoxia when metabolic stress, ROS accumulation, and mitochondrial dysfunction exceed the cell’s adaptive capacity [142] (Figure 4; Table 4). HIF-1α modulates apoptosis in a context-dependent manner, influencing BCL-2 family proteins and contributing to either cell survival or cell death under hypoxic stress [143].

HIF-1α also plays a role in regulating autophagy flux by modulating metabolic pathways such as glycolysis, mitochondrial respiration, and redox adaptation and interacting with other ER-stress regulators, like XBP1, to set thresholds for apoptosis and autophagy, depending on the specific conditions of exposure (Figure 4; Table 4), thereby integrating oxygen availability, ER stress, and metabolic stress into a coordinated cell fate response [59,144].

#### 2.4.5. Nuclear Factor Erythroid 2-Related Factor 2 (NRF2)

NRF2 is a transcription factor that links redox status to the crosstalk between autophagy and apoptosis and functions as a master regulator of the cellular antioxidant response under oxidative and electrophilic stress [145,146]. NRF2 is activated by oxidative stress; it then increases expression of antioxidant genes via antioxidant response elements (ARE), which in turn enhance detoxification and redox buffering capacity and generally support cell survival through induction of genes such as HO-1, NQO1, GCLC, and other cytoprotective enzymes [147] (Figure 4; Table 4).

NRF2 and HIF-1α form an interdependent regulatory loop: NRF2 suppresses HIF-1α by reducing reactive oxygen species (ROS); conversely, HIF-1α suppresses NRF2 via induction of BTB and CNC homology 1 (BACH1) (Figure 4; Table 4), thereby creating a reciprocal regulatory network that coordinates oxidative stress adaptation and hypoxia signaling [148]. Elevated NRF2 levels may paradoxically suppress autophagy under certain contexts by reducing ROS-dependent autophagy signaling and diminishing stress cues that normally activate autophagic machinery [145]. Interestingly, NRF2 also increases p62 expression, which is degraded by autophagy. p62 also acts as a stabilizer of NRF2, creating a feedback loop (Figure 4; Table 4), by binding KEAP1 and sequestering it into autophagosomal structures, thereby preventing KEAP1-mediated ubiquitination and degradation of NRF2 [149,150]. Moreover, NRF2 primarily regulates apoptotic responses indirectly by maintaining redox homeostasis and, in certain contexts, contributes to apoptotic resistance by modulating anti-apoptotic proteins such as BCL-2 [143,151,152].

NRF2 significantly influences autophagy, often through a positive feedback loop involving p62 [153,154]. p62, an NRF2 target gene, binds competitively to KEAP1, liberating NRF2 and further boosting its activation [155]. This p62-KEAP1-NRF2 axis amplifies antioxidant and pro-autophagic responses, crucial for cellular defense [153,154]. NRF2 also directly upregulates various autophagy-related genes, with its activation being essential for autophagy induction in response to diverse stimuli [145]. The role of NRF2 in apoptosis is context-dependent. Generally, NRF2 exerts anti-apoptotic effects by upregulating protective genes and modulating cell death pathways, preventing apoptosis by reducing ROS-induced damage [156,157]. However, in cancer, NRF2 hyperactivation can promote tumor progression and chemoresistance by dampening apoptotic signals, representing a “double-edged sword” effect [158,159]. The p62-KEAP1-NRF2 pathway serves as a central hub for this complex regulatory network, influencing cell fate decisions by integrating oxidative stress signals with both autophagy and apoptosis [155,160].

#### 2.4.6. p53

The p53 protein is an essential cellular mediator of stress responses; it determines whether an autophagic or apoptotic pathway is activated based on the type of stress by integrating signals arising from DNA damage, oxidative stress, oncogene activation, hypoxia, and metabolic imbalance [161,162]. Under less stressful conditions, the p53 protein, when located in the nucleus, induces autophagy by activating autophagy-related genes (e.g., DRAM, ATG4A, SESN2), which support cell survival and repair of cellular damage through transcriptional programs that enhance lysosomal function, autophagosome formation, and AMPK-mediated metabolic adaptation [162,163] (Figure 4; Table 4).

However, under more stressful conditions, the p53 protein, when localized to the cytoplasm, suppresses autophagy by inhibiting the machinery for autophagosome formation, including ULK and its accessory proteins (Figure 4; Table 4), likely through non-transcriptional mechanisms that interfere with autophagy initiation and energy-sensing pathways [63,164]. Furthermore, under more stressful conditions, p53 alters signaling from a pro-autophagy pathway to a pro-apoptosis pathway by transcriptionally activating pro-apoptotic genes such as BCL2-associated X protein (BAX), p53 Upregulated Modulator of Apoptosis (PUMA), and NOXA and by promoting mitochondrial apoptotic priming [161,163] (Figure 4; Table 4).

Moreover, post-translational modifications of p53 further refine its specificity in interacting with target genes and regulatory molecules, including phosphorylation, acetylation, ubiquitination, and methylation, which influence its stability, subcellular localization, and transcriptional selectivity [165,166,167]. Additionally, p53 counteracts NRF2-mediated protective antioxidant responses, promoting apoptosis to eliminate cells beyond repair due to severe damage (Figure 4; Table 4), thereby shifting the balance away from cytoprotective redox adaptation and toward irreversible cell death [168,169].

#### 2.4.7. Signal Transducer and Activator of Transcription 3 (STAT3)

STAT3 is a context-dependent transcription factor that influences both autophagy and apoptosis, particularly in cancer, where it integrates inflammatory, growth factor, and stress-associated signaling pathways [170,171]. STAT3 is generally activated by cytokines (such as IL-6) to regulate genes involved in proliferation and survival through JAK-mediated phosphorylation, dimerization, nuclear translocation, and transcriptional activation of downstream target genes [172,173]. STAT3 exerts anti-apoptotic effects by regulating survival genes such as BCL-2, BCL-XL, and MCL1, thereby promoting cell survival in response to cytokine signaling [174,175].

In several types of cancer, STAT3 is activated and promotes autophagy, a mechanism that enables cancer cells to survive and exhibit therapy-resistant phenotypes; often in association with NRF2 signaling (Figure 4; Table 4), therefore, coupling antioxidant defense with stress-adaptive survival pathways [176,177]. STAT3 can influence autophagy both positively and negatively, depending upon its cellular localization [171,178]. As a nuclear transcription factor, STAT3 can modulate autophagy by regulating the expression of autophagy-related genes (Figure 4; Table 4), either directly or indirectly through transcriptional control of survival, metabolic, and lysosomal regulators [179,180]. Conversely, as a cytoplasmic protein, STAT3 can suppress autophagy by interacting with autophagy suppressors, such as EIF2AK2 and FOXO1 (Figure 4; Table 4), and by interfering with signaling pathways that would otherwise activate the autophagic machinery [171,181]. These two forms of localization underscore the dual, positive and negative, roles that STAT3 can play in autophagy and apoptosis, with nuclear STAT3 often favoring adaptive survival programs and cytoplasmic STAT3 more frequently modulating non-transcriptional restraint of stress responses [182,183].

#### 2.4.8. Nuclear Factor Kappa B (NF-κB)

NF-kB is a central transcription factor that links inflammation, immunity, and cell survival to autophagy–apoptosis pathways by integrating signals from cytokines, pattern-recognition receptors, oxidative stress, and cellular damage [184,185]. The role of NF-kB in promoting or inhibiting autophagy depends on both the cellular context and the stimulus (Figure 4, Table 4), reflecting the complexity of canonical and non-canonical NF-kB signaling pathways [186].

Activation of NF-kB generally promotes anti-apoptotic gene expression, including BCL-2 family members, cIAPs, and FLIP, and regulates autophagy-related genes, either directly or indirectly, through inflammatory and stress-responsive transcriptional programs [187,188]. Previous reports suggest that a reciprocal dependency between Beclin-1 and Atg5 is required for full activation of NF-kB (Figure 4; Table 4), possibly by facilitating scaffold formation and signaling events associated with autophagosomal membranes [189,190]. Interestingly, reports also suggest that autophagy negatively correlates with NF-kB activity in some instances, for example, through degradation of upstream signaling intermediates or limitation of ROS-dependent inflammatory signaling [191,192]. Interestingly, synergy between NRF2 and NF-kB activation can reduce autophagic flux and inflammatory responses by reshaping the cellular redox and inflammatory environment and altering transcriptional outputs that control stress adaptation and lysosomal activity [193,194] (Figure 4; Table 4). Moreover, NF-κB regulates apoptosis primarily through induction of anti-apoptotic genes, including BCL-2 family members, XIAP, and BFL1/A1, contributing to cell survival and inflammatory responses [143,151,152].

## 3. Signal Transduction Pathways in Autophagy–Apoptosis Crosstalk

Cell homeostasis is carefully maintained by a multitude of processes that control the fate of cells; however, apoptosis and autophagy are two critical components of this process [3]. Therefore, it is essential to understand how the complex signal transduction pathways mediating this interaction function, so we can develop specific interventions to promote cell survival or induce cell death, as appropriate [5]. In general, the fine balance between autophagy and apoptosis is mediated by shared signal transducers and regulatory networks, which can result in an adaptive survival response or a programmed death response depending on the magnitude and duration of the stress [5,9].

### 3.1. Unfolded Protein Response (UPR)

**Figure 5:**
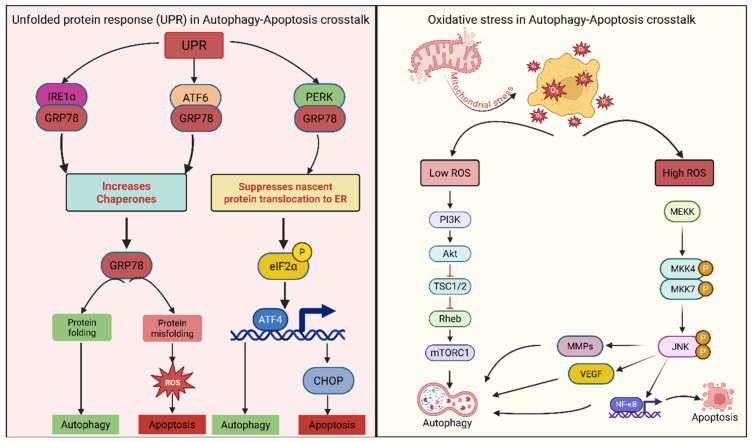
Role of the unfolded protein response (UPR) and oxidative stress in autophagy–apoptosis crosstalk. The **left panel** illustrates the activation of UPR sensors and their connections to autophagy and apoptosis. The **right panel** depicts how different intracellular ROS levels differentially regulate signaling pathways associated with cell survival or cell death.

The unfolded protein response is triggered by ER stress due to the accumulation of misfolded proteins in the ER. Cellular stress, such as nutrient deprivation and hypoxia, disrupts ER homeostasis, leading to the accumulation of misfolded proteins and saturation of the ER protein-folding machinery [195,196] (Figure 5). UPR is an adaptive mechanism that restores ER homeostasis by promoting proper protein folding, reducing protein synthesis, and degrading misfolded proteins through coordinated transcriptional and translational reprogramming [125,196]. However, prolonged or acute ER stress activates pro-apoptotic signaling pathways, leading to cell death when the ER’s adaptive capacity is exceeded [197]. Interestingly, activation of the UPR by mild ER stress typically promotes cell survival by increasing chaperone expression and enhancing ER quality-control mechanisms [198] (Figure 5). During the early adaptive phase, activation of the three canonical ER stress sensors IRE1α, PERK, and ATF6 promotes restoration of proteostasis and cell survival [199]. However, under prolonged or unresolved ER stress, the UPR undergoes a temporal switch from adaptive to terminal signaling, ultimately leading to apoptosis [200].

The transition from an adaptive response to a response that induces cell death is tightly controlled by the ER-resident sensor proteins Inositol-requiring enzyme 1 alpha (IRE1α), Protein kinase RNA-like endoplasmic reticulum kinase (PERK), and Activating transcription factor 6 (ATF6) [124,196,201] (Figure 5). Typically, under normal physiological conditions, these three key UPR sensors are maintained in an inactive state by virtue of their interaction with the chaperone protein Binding immunoglobulin protein/Glucose regulated protein 78 (GRP78/BiP); however, under ER stress conditions, GRP78/BiP dissociates from the UPR sensors, allowing them to become active and initiate the UPR through oligomerization, conformational activation, or translocation-dependent signaling events [125,202,203] (Figure 5).

The PERK pathway suppresses the translation of nascent proteins in the ER to reduce the overall ER workload (Figure 5), primarily by phosphorylating eIF2α, thereby attenuating cap-dependent translation [201]. The ATF6 and IRE1 pathways enhance the ER’s capacity to fold proteins by increasing the production of chaperone and foldase enzymes and improving the efficiency of the ER-associated degradation pathway (Figure 5), which facilitates recognition, retrotranslocation, ubiquitination, and proteasomal degradation of misfolded proteins [203,204]. Furthermore, sustained PERK activation leads to eIF2α phosphorylation, which promotes ATF4 translation rather than global protein synthesis; ATF4 is a transcriptional activator that regulates numerous genes involved in protein folding, amino acid metabolism, and autophagy [59,201,205] (Figure 5). Sustained PERK–eIF2α signaling induces ATF4-dependent expression of CHOP, a key pro-apoptotic transcription factor that promotes cell death by modulating Bcl-2 family proteins and oxidative stress pathways [206].

The PERK arm of the UPR can lead to prolonged phosphorylation of eIF2α via the PERK-eIF2α-ATF4 pathway, resulting in inhibition of protein synthesis and induction of pro-apoptotic proteins such as CHOP when ER stress becomes persistent or irreparable [201] (Figure 5). In addition, the IRE1α and ATF6 arms of the UPR regulate protein folding and degradation primarily by transcriptionally upregulating chaperone proteins, including GRP78, GRP94, and PDI, as well as ERAD-associated genes that enhance proteostasis capacity [124]. Concurrently, hyperactivation of IRE1α triggers regulated IRE1-dependent decay (RIDD) and activation of stress kinases such as JNK, further amplifying apoptotic signaling [206]. Thus, the balance between the adaptive and terminal UPR phases is a critical determinant of cell fate under ER stress [207]. Therefore, the UPR can reduce the protein-folding burden on the ER and promote cellular survival when ER stress is removed. However, if ER stress remains unresolved, the UPR can also become cytotoxic and may initiate apoptosis via several mechanisms, including increased generation of ROS and activation of caspase-dependent apoptotic signaling, along with mitochondrial dysfunction, calcium dysregulation, and transcriptional induction of pro-death mediators [208,209,210]. ATF4 can drive the expression of genes involved in oxidative stress responses, autophagy, amino acid metabolism, and apoptosis, particularly by inducing CHOP-mediated cell death and by shifting the balance from adaptive stress responses toward terminal apoptotic signaling under sustained ER stress [118,211] (Figure 5).

In the context of cancer, tumor cells frequently exploit the adaptive arm of the UPR to survive hostile microenvironmental conditions such as hypoxia, nutrient deprivation, and therapeutic stress [207,212]. For instance, the IRE1α–XBP1 signaling axis has been shown to promote tumor cell survival, proliferation, and aggressiveness across multiple cancer types [213,214]. Additionally, activation of IRE1α-mediated XBP1 splicing confers pro-survival advantages and contributes to therapy resistance in cancer cells [215]. Similarly, PERK signaling supports tumor adaptation to hypoxic stress and facilitates metabolic reprogramming, thereby enhancing tumor progression and survival [215]. Cancer cells often maintain a chronically activated, “hyper-adaptive” UPR state that enables them to tolerate persistent stress and evade apoptosis [216]. However, excessive or sustained ER stress can push cancer cells toward terminal UPR activation, which can be therapeutically exploited to induce apoptosis [200]. This dual role of the UPR supporting tumor survival in its adaptive phase while promoting cell death under prolonged stress highlights its significance as both a driver of cancer progression and a promising therapeutic target [217,218].

### 3.2. Oxidative Stress

The relationship between redox homeostasis and autophagy–apoptosis is directly linked to treatment options in cancer therapy, as oxidative stress strongly influences whether cancer cells adapt, survive, or undergo cell death in response to therapy [219,220]. Regulation of redox balance affects the immune system and, in turn, the tumor microenvironment by altering inflammatory signaling, immune cell recruitment, cytokine production, and stromal interactions [221]. Due to their high reactivity, ROS molecules are closely associated with multiple forms of programmed cell death, such as apoptosis, ferroptosis, necroptosis, and autophagy through oxidative modification of lipids, proteins, DNA, and signaling molecules [222,223] (Figure 5).

A shift towards higher ROS production than antioxidant activity is a common feature in many cancers, leading to alterations in protein quality, signal transduction, and gene expression, as well as mitochondrial and ER dysfunction [224]. A shift in redox status affects cancer cells’ ability to proliferate, metastasize, invade, and die [225] (Figure 5). Low concentrations of ROS can stimulate proliferation in tumor cells through pathways such as PI3K/Akt/mTOR and RAS/Raf/MAPK, whereas high concentrations can induce apoptosis and autophagy and inhibit tumor growth by overwhelming antioxidant defenses and activating stress-responsive death pathways [177,226] (Figure 5). These findings demonstrate the importance of identifying the type and concentration of ROS when planning therapeutic interventions, as different ROS can produce distinct effects on cellular pathways, for example, superoxide, hydrogen peroxide, and hydroxyl radicals differ in diffusion capacity, reactivity, and subcellular targets [227,228]. Intermediate concentrations of ROS can stimulate protective autophagy, which protects the cell from oxidative damage and supports cell viability by removing damaged organelles and proteins, indicating the dose- and context-dependent roles of ROS in determining cell fate (Figure 5), particularly through mitophagy and the degradation of oxidized macromolecules [229,230]. ROS has been identified as a stimulus for several important oncogenic signaling pathways, such as Mitogen-activated protein kinase (MAPK) and Phosphoinositide 3-kinase (PI3K)/Phosphatase and tensin homolog (PTEN), which can contribute to tumor growth and survival by regulating key proteins and transcription factors, such as NF-κB (Figure 5), through reversible oxidation of phosphatases, kinases, and redox-sensitive transcriptional regulators [177,231]. ROS are often elevated in cancer cells due to changes in mitochondrial metabolism, hypoxia, and oncogene activation, leading to DNA damage, genomic instability, and oxidative modification of key signaling proteins involved in tumorigenesis [224,232] (Figure 5).

The dual role of ROS as both pro- and anti-tumorigenic is heavily dependent on ROS concentration and the stage of cancer progression. ROS in early-stage cancers promotes tumor initiation through mutagenic effects on proto-oncogenes and tumor suppressor genes by inducing DNA strand breaks, base modifications, and replication-associated errors [177,233] (Figure 5). However, chronic oxidative stress in advanced tumors promotes tumor progression and drug resistance through the activation of adaptive signaling pathways, such as PI3K/Akt and NF-κB, that enhance cell survival and proliferation (Figure 5), and through the selection of cells with enhanced antioxidant and metabolic adaptability [234]. Conversely, although elevated ROS levels can promote tumorigenesis and metastasis by activating pro-survival signaling, extremely high ROS levels can elicit anti-tumorigenic effects, such as cell death, cell cycle arrest, and senescence, by causing irreparable macromolecular damage and activating checkpoint and death signaling pathways [235]. The fine-tuning of ROS production is important, as it is an activator of pro-survival signaling pathways such as MAPK/ERK1/2, p38, JNK, and PI3K/Akt, which ultimately promote angiogenesis, metastasis, and survival when they interact with NF-κB, MMPs, and VEGF (Figure 5), thereby linking oxidative stress to tumor progression, extracellular matrix remodeling, and vascular adaptation [236].

### 3.3. MAPK Pathway

**Figure 6:**
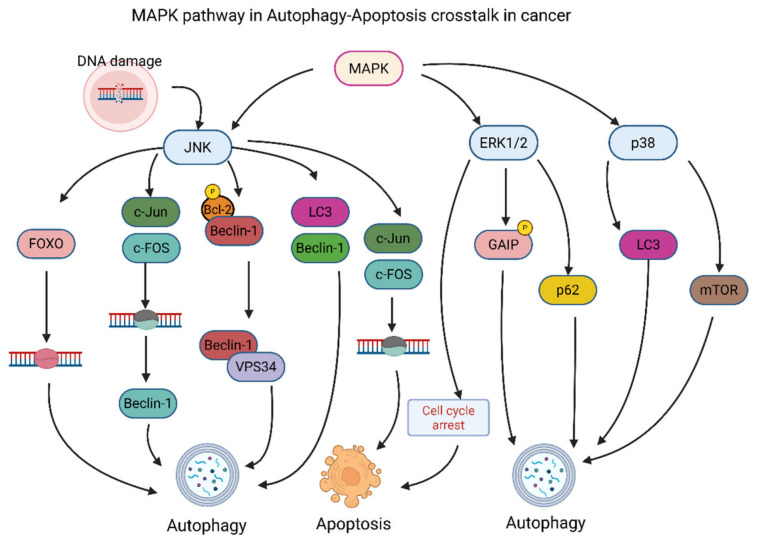
Role of mitogen-activated protein kinase (MAPK) proteins in autophagy–apoptosis crosstalk in cancer. Figure 6 illustrates how different kinases within the MAPK pathway regulate autophagy and apoptosis through distinct molecular signaling mechanisms.

Sustained ERK activation, seen in many cancers, promotes cancer cell survival and prevents programmed cell death through several mechanisms (Figure 6), including maintenance of proliferative signaling, inhibition of pro-apoptotic mediators, and modulation of autophagy-associated proteins [237,238]. Chronic JNK activation, found in cancers such as triple-negative breast cancer, promotes cancer progression through autophagy and metabolic changes by supporting stress adaptation, mitochondrial remodeling, and survival under hostile tumor microenvironmental conditions [239]. In contrast, although p38 MAPK typically transmits pro-apoptotic signals, it can also induce autophagy to provide energy for senescent cells and support survival under metabolic or genotoxic stress [240] (Figure 6).

The activated form of JNK can disrupt the interactions between Bcl-2 and Beclin-1 by phosphorylating Bcl-2 family proteins, thereby allowing Beclin-1 to bind to Vacuolar protein sorting 34 (Vps34) and form the PI3KIII complex that leads to the formation of autophagosome and initiation of autophagosome nucleation [189,241] (Figure 6). Additionally, activated JNK can phosphorylate and activate the transcription factors c-Jun and c-Fos, which, in turn, activate Beclin-1 to initiate autophagy through AP-1-dependent transcriptional programs [189]. In contrary, activation of c-Jun and c-Fos by JNK also activates cell death by inducing pro-apoptotic and stress-responsive genes when cellular damage is severe or prolonged [242] (Figure 6). Furthermore, activated JNK also activates a variety of downstream targets such as FOXO transcription factors that are responsible for regulating the expression of many of the Autophagy-related genes (ATGs), including VPS34, ATG12, LC3, BNIP3, ULK2, ATG4b, and ATG12L, which are involved in the initiation and progression of autophagy (Figure 6), thereby linking stress signaling to the transcriptional machinery that sustains autophagic flux [243].

While the ERK1/2 pathway is most often associated with promoting cell proliferation, it can also influence autophagy, and its effects may depend on the specific cancer type, the cellular environment (Figure 6), and the strength and duration of upstream growth factor signaling [56]. For example, ERK can inhibit autophagy by phosphorylating cytoplasmic signaling proteins, such as G alpha-interacting protein (GAIP), and/or nuclear components, such as ternary complex factor transcription factors, thereby facilitating cell survival and maintaining pro-survival transcriptional and metabolic programs [244] (Figure 6). On the other hand, p38 MAPK can modulate autophagy by modifying autophagy-related proteins, such as LC3 and Atg5, and it can also interact with the Wnt/β-catenin pathway via Glycogen synthase kinase 3 beta (GSK3β) (Figure 6), thereby integrating stress signaling with developmental and metabolic regulatory networks [245]. The complexity of these pathways underscores the necessity of developing a complete understanding of how they intersect and diverge to control cellular fate and presents an opportunity to develop targeted therapeutic approaches.

The JNK-MAPK pathway can regulate autophagy at the transcriptional level by modulating the transcription of key regulatory genes such as LC3, Beclin-1, Sestrin2, and ATG5/7, or at the non-transcriptional level by phosphorylating Bcl-2 (Figure 6), thereby rapidly releasing Beclin-1 from inhibitory complexes [53]. The JNK is part of the MAPK family and regulates numerous cellular functions, including cell proliferation and apoptosis. The JNK-signaling cascade is often activated in response to DNA damage and can cause apoptosis, and/or autophagy, and/or necroptosis depending on the extent of damage and the balance between adaptive and death-promoting downstream effectors [57] (Figure 6). Furthermore, JNK has been shown to induce autophagy by producing ROS, thereby activating ASK1, which in turn activates additional JNK and deactivates phosphatase activity that would otherwise inhibit JNK [64] (Figure 6), creating a positive feedback loop that amplifies stress signaling [71]. JNK’s involvement in these two modes of regulation underscores its central role in determining cell fate and, as such, supports the need to specifically regulate JNK as a therapeutic target in cancer [57].

Like JNK, P38 MAPK has been identified as a key regulator of cell fate and similar to JNK, regulates both apoptosis and autophagy in response to DNA damage [57,246] (Figure 6). In particular, it has been demonstrated that P38 MAPK phosphorylates ATG5 at threonine-7; this modification is necessary for the formation of autophagosomes and contributes to early autophagy machinery activation under stress conditions [245] (Figure 6). The phosphorylation of ATG5 by P38 MAPK leads to the conversion of LC3-I to LC3-II, which is a key event in the elongation and maturation of the autophagosomal membrane through enhancement of autophagosome biogenesis and membrane dynamics [247] (Figure 6). The ability of P38 MAPK to interact with multiple autophagy-related proteins underscores its role in regulating cellular responses to stress while balancing cell survival and cell death pathways [248]. Beyond direct phosphorylation, P38 MAPK can also modulate autophagy indirectly by regulating multiple upstream kinases and phosphatases, which ultimately affect mTOR signaling, a major negative regulator of autophagy and thereby influence whether autophagy proceeds as a cytoprotective or cytotoxic process [240] (Figure 6).

### 3.4. Calcium Signaling

Calcium is a second messenger that plays a critical role in modulating the crosstalk between autophagy and apoptosis, determining whether a cell lives or dies in a malignant environment by regulating mitochondrial metabolism, ER stress responses, lysosomal signaling, and activation of calcium-dependent enzymes [249,250]. Cancer cells exhibit alterations in their ability to regulate calcium signaling that promote tumor cell growth and invasion and confer resistance to therapy by disrupting calcium homeostasis within the cell and by rewiring intracellular Ca^2+^ transfer between the ER, mitochondria, and lysosomes. Therefore, calcium signaling has the capability to function as an “on/off” type regulatory system in determining the fate of a stressed cell, in which calcium signal act as a “rheostat” and allow the cell to make the choice to undergo either autophagic survival or apoptotic commitment depending upon the environmental stress [250,251].

**Figure 7:**
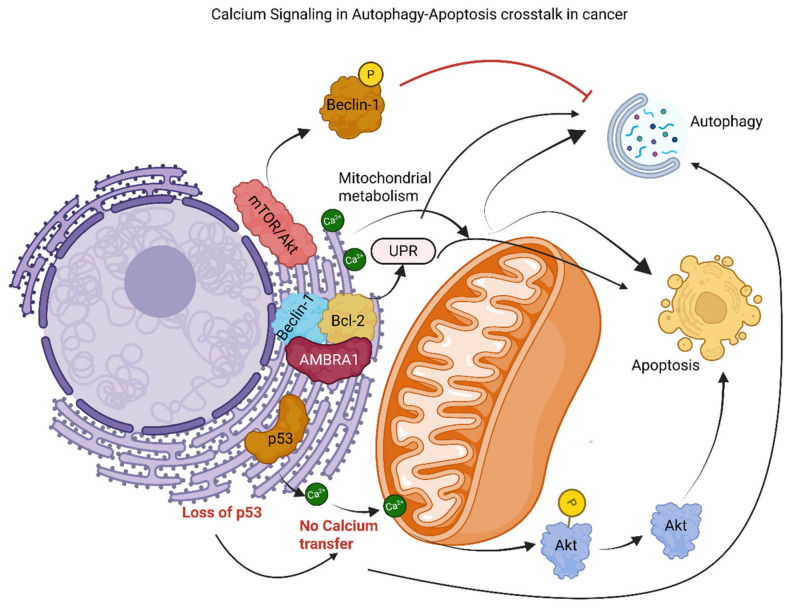
Role of calcium signaling in autophagy–apoptosis crosstalk in cancer. Figure 7 illustrates the dual role of calcium signaling in activating autophagy and apoptosis. The black arrow indicates activation, and the red line indicates inhibition.

The influence of calcium regulation in both the mitochondria and the endoplasmic reticulum was identified, along with the roles of several proteins, including calpains, calmodulin, and the Bcl-2 family (Figure 7), all of which act as calcium-sensitive effectors that translate Ca^2+^ flux into survival or death signaling [252]. Additionally, there are now increasing amounts of data that illustrate the multifunctional nature of proteins that bind Ca^2+^ and those that facilitate Ca^2+^ transport in modulating cell fate in cancerous states [249] (Figure 7). Specifically, the IP3 receptor facilitates Ca^2+^ release from the endoplasmic reticulum and thereby influences mitochondrial bioenergetics and both apoptosis and autophagy in cancer cells by governing calcium transfer at mitochondria-associated membranes (MAMs) [250,253] (Figure 7). Also, Inositol 1,4,5-trisphosphate receptor (IP3R)-mediated Ca^2+^ release supports the maintenance of mitochondrial metabolic processes required for cancer cells’ anabolic functions and modulates autophagic flux under stress; these two processes are important for determining tumor progression and the potential for programmed cell death, because mitochondrial Ca^2+^ uptake stimulates TCA cycle dehydrogenases and ATP production, whereas reduced transfer can activate AMPK and autophagy [254,255] (Figure 7). Additionally, cancer cells can also modify their own use of the machinery involved in IP3R-mediated Ca^2+^ flux to survive under therapeutic insults (Figure 7). For example, oncogenic pathways that include upregulation of FBXL2 after PTEN inactivation can downregulate IP3R activity, decreasing mitochondrial Ca^2+^ loading and maintaining cancer cell viability by limiting mitochondrial calcium overload and preventing apoptosis [256,257]. On the other hand, PTEN can inhibit the upregulation of FBXL2 and increase Ca^2+^ flux from the IP3R3 receptor to mitochondria, thereby restoring the sensitivity of cancer cells to apoptosis and limiting their proliferation by re-establishing pro-apoptotic ER–mitochondrial calcium transfer [256] (Figure 7).

p53 that relocates to the endoplasmic reticulum during stress also modulates IP3R3-mediated Ca^2+^ transfer at mitochondria-associated membranes (MAMs) to potentiate Akt dephosphorylation via Promyelocytic leukemia protein (PML) recruitment and to thereby potentiate apoptosis while limiting autophagy-based survival in cancer cells by enhancing constitutive calcium release toward mitochondria and favoring mitochondrial permeabilization under severe stress [250,258] (Figure 7). At the same time, Akt-mediated suppression of IP3R3 activity reduces ER-to-mitochondria Ca^2+^ transfer, thereby enhancing cancer cell resilience to genotoxic insults by simultaneously reducing apoptosis susceptibility and increasing autophagic flux through reduced mitochondrial ATP generation and compensatory activation of AMPK-dependent autophagy pathways [259,260] (Figure 7). Furthermore, MAM dysfunction implicates BRCA1, which, when wild type, binds to and activates IP3Rs to maintain apoptosis sensitivity; however, mutations in BRCA1 disrupt this interaction, leading to reduced Ca^2+^-mediated autophagy–apoptosis crosstalk and increased resistance to chemotherapy [250,261] (Figure 7).

Additionally, mTORC2/Akt complexes are localized to MAMs, phosphorylate and inhibit Beclin-1, a protein that interacts with IP3R3 and is bound by Bcl-2, thereby inhibiting autophagy and promoting cancer cell survival under nutrient-limited conditions while also reinforcing survival signaling at the ER–mitochondria interface [250,262] (Figure 7). On the other hand, AMBRA1 binds to both Bcl-2 and Beclin-1 at MAMs to regulate Ca^2^-dependent ER stress through the UPR; thus, it dictates whether the cell will undergo autophagy for adaptation or commit to apoptosis by influencing the availability of Beclin-1 and the intensity of ER stress signaling [263] (Figure 7). The regulation of Ca^2+^ exchange between the ER and mitochondria by the regulators, including but not limited to IP3R3, PML, PTEN, and p53, is fundamental to the autophagy–apoptosis crosstalk, where increased linkage of the ER to mitochondria promotes Ca^2^-driven apoptosis while decreased linkage favors survival of the cell and enhanced mitochondrial respiratory function in cancer cells depending on whether calcium transfer reaches bioenergetic or pro-death thresholds [258,264] (Figure 7). The absence of either PML or p53 in cancer cells diminishes the amount of Ca^2+^ transferred from the ER to the mitochondria via IP3R, resulting in the activation of AMPK through the mTOR/Ulk-1 pathway; this results in stimulation of pro-survival autophagy that allows continued proliferation of cancer cells and confers resistance to therapeutic drugs such as 5-fluorouracil by shifting cellular metabolism toward stress adaptation rather than mitochondrial apoptosis [63,265] (Figure 7). Furthermore, disruption of the p53-PML interaction at ER-mitochondria interfaces decreases constitutive ER-Ca^2+^ release, thereby reducing mitochondrial respiration and ATP generation, activating AMPK and stimulating autophagy, enhancing tumor adaptation to metabolic stress and damage caused by therapeutic intervention [266] (Figure 7). Additionally, lysosomal TRPML1 channels mobilize Ca^2+^ to activate Ca^2+^/calmodulin-dependent protein kinase kinase beta (CaMKKβ)-AMPK signaling and ULK1/VPS34 complexes to initiate phagophore formation and autophagosome biogenesis, respectively, thereby supporting autophagic adaptation in cancer cells, independently of IP3R-mediated mitochondrial fluxes and linking lysosomal calcium release directly to the early autophagy machinery [250,267]. Therefore, an advantage of modulating lysosomal TRPML1 channels for therapeutic purposes is that it can disrupt the survival mechanisms of cancer cells that rely on autophagy, thereby potentially altering Ca^2+^-mediated degradation pathways and leading to apoptosis [268]. Furthermore, simultaneous activation of the UPR via PERK signaling at these Ca^2+^ dysregulated lysosomal interfaces can increase communication between autophagic responses to ER stress and the suppression of oxidative metabolic activity and promotion of apoptosis in tumor cells by integrating calcium imbalance, translational stress, and mitochondrial dysfunction into a coordinated cell fate response [250,264] (Figure 7).

## 4. Natural Products and FDA-Approved Drugs in Modulating Autophagy–Apoptosis Crosstalk

The complex interaction between autophagy and apoptosis in cancer cells is an area of emerging therapeutic interest, as it can be harnessed either to enhance tumor cell death through the synergistic action of both processes or to reduce cell death through autophagy-induced survival [269]. Approximately 47% of all antitumor agents are derived from natural compounds, which have demonstrated low toxicity and multitarget activity [270]. The use of natural compounds as modulators of autophagy and apoptosis highlights their potential to leverage autophagy’s dual role in cancer, either inhibiting pro-survival autophagy or activating cytotoxic forms of autophagy to improve treatment options for patients with advanced cancers who have developed therapeutic resistance [271]. Additionally, the use of phytochemicals offers a complementary strategy to traditional chemotherapy by pharmacologically targeting the autophagy–apoptosis signaling pathway at the molecular level, providing enhanced selectivity and specificity towards killing cancer cells [272].

### 4.1. Natural Compounds

Phytochemicals are currently being investigated as potential therapeutic complements to traditional cancer treatments that target the autophagic-apoptotic pathway. Phytochemicals have been identified to trigger apoptosis using two major mechanisms of action. The first mechanism of action of phytochemicals has been to disrupt mitochondrial function, thereby activating caspase-9 and ultimately triggering intrinsic programmed cell death, known as apoptosis, through mitochondrial outer membrane permeabilization, cytochrome c release, and apoptosome formation [273]. A second mechanism of action for phytochemicals involves the activation of caspase-8 through their interaction with FAS ligand, a receptor that initiates the extrinsic apoptotic pathway and promotes death receptor-mediated apoptotic signaling [250,274]. In addition to inducing apoptosis, some phytochemicals have been shown to trigger ER stress, a condition that is also linked to apoptosis. Studies examining the anticancer effects of many phytochemicals suggest they may shift the balance between autophagy and apoptosis [273,275]. Many studies have demonstrated that compounds such as curcumin and berberine can activate AMPK while simultaneously inhibiting PI3K/protein Akt/mTOR signaling pathways, thereby increasing apoptosis in non-small-cell lung cancer cells [69,219] and favoring metabolic stress-associated autophagic responses that can become cytotoxic under sustained treatment conditions [69,276]. Other studies have demonstrated that BH3 mimetic agents such as gossypol and obatoclax can promote autophagic-mediated necroptosis, while promoting apoptosis in other forms of cancer by disrupting anti-apoptotic Bcl-2 family interactions and altering mitochondrial and autophagic signaling thresholds [277].

In addition to apoptosis, natural compounds can induce other forms of programmed cell death, including necroptosis, pyroptosis, ferroptosis, and cuproptosis. There is growing interest in ferroptosis as a form of programmed cell death that could be used to treat cancer, given its role in reducing glutathione and increasing oxidative damage via iron-dependent lipid peroxidation [278,279]. The fact that natural compounds can modulate multiple forms of programmed cell death presents a significant opportunity for future research; however, their use in humans is limited by their very low solubility and poor bioavailability [280,281]. Natural compound nanoparticles can be designed to improve the solubility and bioavailability of poorly soluble drugs and to provide targeted delivery to specific tissues or organs within the body, while potentially reducing off-target toxicity and improving pharmacokinetic stability [282].

Natural products containing a wide variety of phytochemicals, including polyphenols and alkaloids, can significantly modulate autophagy–apoptosis interactions by leveraging their antioxidant capabilities to both inhibit and stimulate cytostatic and cell death functions in cancerous cells depending on the redox context, tumor type, and intracellular stress burden [283]. These compounds can trigger the ER stress pathway, promote apoptosis while counter the protective role of autophagy in maintaining ER homeostasis, thereby shifting the UPR from an adaptive to a pro-death program [2]. In addition, some compounds, such as Resveratrol, have been shown to increase the level of pro-apoptotic protein Bax and the autophagy-related protein Beclin-1, while decreasing the activity of the anti-apoptotic proteins c-Myc and PI3K/Akt, thus simultaneously inducing apoptosis and autophagic cell death in NSCLC models such as A549 cells through coordinated disruption of survival and metabolic signaling networks [69,284,285]. Likewise, Shikonin has been shown to increase ROS levels in cancerous cells beyond those in normal cells, leading to disruptions in mitochondrial function and suppression of PI3K/Akt/mTOR, which, in turn, triggers apoptosis, autophagy, and ferroptosis in lung malignancies by overwhelming antioxidant defenses and promoting oxidative damage-driven cell death pathways [286,287,288]. Quercetin and Anthocyanin share similar capabilities to alter intracellular Ca^2+^ homeostasis by regulating the plasma membrane Ca^2+^-ATPase, ultimately enhancing ferroptosis-apoptosis interactions in cancerous tissues through disruption of calcium-dependent survival and mitochondrial signaling pathways [289,290].

The modulation of cellular homeostasis by natural products such as alkaloids and terpenoids is a strategic approach that not only avoids tumor drug resistance but also offers the potential for precise autophagy regulation across a wide range of malignancies [291,292]. The various chemical structures present in natural products enable modulation of multiple signaling cascades, including the PI3K/Akt/mTOR and MAPK pathways, thereby allowing simultaneous inhibition of tumor angiogenesis and enhancement of autophagic flux toward cytotoxic outcomes in preclinical breast cancer models, increasing the likelihood of multi-target antitumor efficacy [293,294]. Ginsenoside K is a multi-target modulator that activates the AMPK/mTOR and JNK pathways, increasing Microtubule-associated protein 1 light chain 3 (LC3-II) and Beclin-1 levels while decreasing p62 levels, thereby promoting autophagy-mediated apoptosis in NSCLC cell lines such as A549 and H1975 by simultaneously inducing autophagic flux and stress-associated apoptotic signaling [69,295]. Ginsenoside K also disrupts the ATG6-Bcl-2 interaction and increases JNK signaling activity to enhance autophagic flux and vesicle accumulation, ultimately reducing cell survival when JNK is silenced [180], indicating that JNK signaling is mechanistically required for its full cytotoxic effect [270,296].

Similarly, Voacamine enhances the cytotoxicity of Doxorubicin in multidrug-resistant osteosarcoma cells by promoting autophagy-dependent cell death, independent of apoptosis, and may help overcome chemoresistance by bypassing defective apoptotic machinery [297]. Tetrandrine also induces dose-dependent apoptosis in a wide range of cancer cells, including breast, liver, leukemia, colon, and pancreatic cancers, by perturbing the equilibrium between JNK and ERK signaling and shifting stress signaling toward pro-death outputs [298,299]. Similarly, Harmine has been shown to induce autophagy and apoptosis in gastric cancer cells by inhibiting Akt/mTOR/p70S6K signaling and increasing Beclin-1 expression, in conjunction with mitochondria-mediated caspase activation, thereby coupling autophagy induction with intrinsic apoptotic execution [300]. Indole Alkaloids, such as Chaetoglobosin G and Bisleuconothine A, have been identified as being capable of targeting interconnected autophagy networks involving MAPKs, PI3K/Akt/mTOR, and JAK2/STAT3 pathways to exert antitumor effects in a wide variety of cancerous tissues through broad modulation of growth, stress, and inflammatory signaling pathways [301,302]. Evodiamine, an indole alkaloid, demonstrates this capability by increasing the release of calcium ions from the endoplasmic reticulum and activating JNK signaling to induce autophagy, while simultaneously activating calcium channels in mitochondrion-mediated apoptosis in glioblastoma cells, thereby linking ER calcium dysregulation to mitochondrial death signaling [302,303]. Baicalein demonstrates a similar dual modulation of cellular processes by down-regulating the PI3K/Akt/mTOR pathway to increase autophagy, as evidenced by elevated LC3-II/LC3-I and Beclin-1 levels, and to promote apoptosis in A549 NSCLC cells [69,304]. Piperlongumine disrupts the PI3K/Akt/mTOR axis by decreasing p-Akt and p-mTOR levels, thereby promoting apoptosis while inhibiting autophagy to limit cancerous cell proliferation, illustrating that phytochemical-induced autophagy can be either pro-survival or pro-death depending on the molecular context [305].

Hepatocellular carcinoma cells (HepG2) treated with EGCG show reduced cell growth by decreasing the secretion of the tumor marker α-fetoprotein (AFP). EGCG induces autophagy by promoting the conversion of LC3-I to LC3-II and increasing autophagosome formation. EGCG directly interacts with LC3 and promotes LC3-II conversion, thereby enhancing the degradation of AFP aggregates via autophagy. This shows that EGCG can enhance autophagy and contribute to the suppression of hepatocellular carcinoma progression [306].

Honokiol, a polyphenol found in *Magnolia officinalis*, induces both apoptosis and autophagy. It does so by increasing intracellular ROS and activating the ERK signaling pathway, thereby leading to G0/G1 cell cycle arrest. This activation promotes both apoptosis, as indicated by caspase activation and mitochondrial dysfunction, and autophagy, as indicated by increasing LC3-II and ATG proteins. Moreover, it suppresses tumor growth by ROS-mediated ERK activation, which triggers autophagy and apoptosis in osteosarcoma cells [307].

Luteolin, a flavonoid found in plants such as celery and thyme, induces mitochondrial-mediated apoptosis in glioblastoma cells by increasing BAX, activating caspase-3/7, and promoting PARP cleavage. It triggers autophagy, as indicated by the LC3-I to LC3-II conversion and autophagosome formation. Inhibition of autophagy with 3-methyladenine (3-MA) enhances luteolin-induced apoptosis, suggesting that luteolin-induced autophagy acts as a cell-survival mechanism in glioblastoma cells [308].

Moreover, phytochemicals can trigger autosis, a unique type of autophagy-dependent cell death characterized by an inability to undergo apoptosis, in cancer cells by activating both oncogenic and stress-responsive signaling pathways, thereby elevating persistent autophagic flux beyond the cytoprotective threshold [309]. Several phytochemicals, such as curcumin, resveratrol, berberine, quercetin, celastrol, and withaferin A, have been shown to inhibit the PI3K/AKT/mTOR pathway, which is generally considered to inhibit autophagy [310]. As a consequence of inhibiting this pathway, ULK1 activity is no longer suppressed by mTOR, resulting in enhanced autophagosome formation [311]. Additionally, many of these phytochemicals activate AMPK, which in turn promotes autophagy through both direct and indirect mechanisms, including phosphorylation of ULK1 and inhibition of mTORC1 [312]. An increase in reactive oxygen species (ROS) levels by phytochemicals activates JNK, p38 MAPK, and ER stress signaling. Activation of these signaling pathways leads to transcriptional and post-transcriptional increases in the expression of autophagy-associated proteins, such as ATG5, ATG7, Beclin-1, and LC3 [273,313]. Ultimately, continued elevation in ROS levels, along with unresolved ER stress, may lead to increased autophagic signaling via two additional pathways: PERK/eIF2α/ATF4/CHOP and IRE1/JNK [314,315]. A list of natural products targeting the autophagy–apoptosis crosstalk is presented in Table 5.

### 4.2. FDA Approved Drugs

FDA-approved drugs, such as sorafenib have been shown to modulate crosstalk between autophagy and apoptosis and can be combined with wogonin to reduce autophagy and increase apoptosis, thereby reducing growth in hepatocellular carcinoma models through simultaneous suppression of survival signaling and enhancement of mitochondrial apoptotic pathways [316]. Chloroquine, an FDA-approved drug originally developed as an antimalarial but now being repurposed for the treatment of various types of cancer, blocks the late stages of autophagy by preventing lysosomal acidification, thereby increasing the amount of apoptosis induced in many different cancers by creating an accumulation of autophagosomes and enhancing the synergy of chemotherapeutic agents through disruption of autophagosome–lysosome fusion and lysosomal degradative capacity [317,318]. Additionally, hydroxychloroquine, another FDA-approved antimalarial, has been shown to disrupt autophagic flux when combined with chemotherapy agents such as temozolomide, gemcitabine, and bortezomib in phase I/II trials in glioblastoma, pancreatic cancer, and multiple myeloma, thereby increasing apoptosis and sensitizing tumor cells to treatment-induced stress [319,320,321,322]. In addition, a plethora of research shows that several drugs already approved by the FDA can be combined with other agents to modulate the crosstalk between autophagy and apoptosis. For example, metformin, an FDA-approved diabetes drug, has been shown in preclinical studies to sensitize cancer cells to apoptosis by activating AMPK and inhibiting mTOR signaling, thereby modulating the crosstalk between autophagy and apoptosis and shifting cellular metabolism toward energy stress [323]. In addition, it has been demonstrated that sorafenib, an FDA-approved anticancer agent, can both stimulate autophagy by increasing LC3 and Beclin-1 expression and promote apoptosis via mitochondrial pathways in hepatocellular carcinoma cells, illustrating its dual capacity to trigger adaptive autophagy and pro-death signaling [324]. The combinatorial treatment with the potent autophagy blocker hydroxychloroquine and the kinase inhibitor sorafenib has been shown to enhance apoptotic effects by preventing autophagy-mediated stress adaptation [319]. In addition, the combination of sorafenib and vorinostat has been shown to inhibit autophagy by acetylating Beclin-1 and to synergistically promote apoptosis and cell cycle arrest in hepatocellular carcinoma models through epigenetic and post-translational modulation of survival pathways [325,326].

Furthermore, it has been demonstrated that everolimus, which is an FDA-approved mTOR inhibitor, can synergize with angiogenesis inhibitors such as tivozanib to slow the progression of refractory metastatic colorectal cancer in patients and that this effect is associated with the induction of apoptosis through the DR5/Fas-associated protein with death domain/Caspase-8 axis, and that the addition of chloroquine to block autophagy can enhance this effect in clinical trials by suppressing compensatory survival autophagy [327]. It has also been demonstrated that temsirolimus, an FDA-approved mTOR inhibitor, can synergize with hydroxychloroquine in phase I trials in patients with metastatic prostate cancer, thereby enhancing docetaxel-induced apoptosis by blocking autophagy flux and increasing treatment-associated cytotoxic stress [328]. Moreover, bortezomib, an FDA-approved proteasome inhibitor, has been shown to regulate crosstalk between autophagy and apoptosis by blocking protective autophagy in lymphoma models and by enhancing apoptotic cell death through interactions with mTOR pathway modulators while also increasing proteotoxic stress within tumor cells [329,330]. Temozolomide, an FDA-approved chemotherapeutic agent, has been shown in Phase I clinical trials to act synergistically with Dasatinib, promoting autophagy to enhance temozolomide’s effectiveness and inducing apoptosis in resistant cells, indicating that, in some contexts, autophagy can facilitate therapeutic cytotoxicity rather than resistance [331,332]. Doxorubicin, an FDA-approved anthracycline, has been shown to exhibit synergy with either Pantoprazole or Hydroxychloroquine in phase I clinical trials in patients with advanced solid tumors and lymphoma, inhibiting protective autophagy during chemotherapy and creating a condition in which apoptosis can occur through impairment of lysosomal buffering and stress adaptation mechanisms [333,334,335]. Rapamycin, a well-known FDA-approved mTORC1 inhibitor, has also been shown to modulate autophagy–apoptosis crosstalk in breast cancer by synergizing with Resveratrol to increase autophagic flux and kill more tumor cells via apoptosis, demonstrating that induction of autophagy can also contribute to cytotoxic outcomes depending on the treatment context [336,337].

Additionally, imatinib, an FDA-approved tyrosine kinase inhibitor, has been shown to induce cytotoxicity when combined with a late-stage autophagy inhibitor, such as Bafilomycin A1, in malignant glioma cells and to induce apoptosis via the formation of autophagosomes followed by blockade of their clearance, which enhances cellular stress [29]. Furthermore, Carfilzomib, an FDA-approved proteasome inhibitor, has been shown to synergize with autophagy inhibitors such as Chloroquine or Hydroxychloroquine in myeloma models, modulating autophagy–apoptosis crosstalk and improving anti-tumor efficacy through simultaneous inhibition of proteasomal and lysosomal stress-relief pathways [338,339]. GANT61, a Hedgehog signaling pathway inhibitor, was also shown to induce autophagy-dependent cytotoxicity in hepatocellular carcinoma cells; these effects were partially reversed by Chloroquine or 3-Methyladenine, demonstrating the context-dependent nature of autophagy and apoptosis in HCC cell lines and underscoring that autophagy may either support or impair tumor cell survival depending on the signaling context [340].

Metformin triggers autophagy in colorectal cells by activating AMPK and further blocking down the mTOR pathway. This process increases autophagy markers such as LC3B and can further affect cancer stem cell activity. When metformin is used alongside other treatments, such as chemotherapy agents (e.g., 5-fluorouracil, oxaliplatin, irinotecan, or doxorubicin), radiotherapy, or immunotherapy, metformin-induced autophagy may promote cancer cell death by enhancing apoptosis [341]. In A549 lung cancer cells, gefitinib treatment increases autophagy and promotes apoptosis, thereby further reducing cell proliferation by inhibiting the PI3K/AKT/mTOR pathway. Treatment with Gefitinib results in decreased expression of P13K, AKT, and mTOR, as well as their phosphorylated forms, thereby promoting autophagy and apoptotic cell death. Hence, gefitinib suppresses tumor growth by inducing autophagy and apoptosis through inhibition of the PI3K/AKT/mTOR pathway [342]. In addition to lung cancer, gefitinib has also been shown to induce autophagy in breast cancer cells. Treatment with gefitinib increases the autophagic flux in both gefitinib-sensitive and insensitive or resistant breast cancer cell lines by downregulation of AKT and ERK signaling pathways. Attenuation of advanced-stage autophagy by hydroxychloroquine or bafilomycin A1 treatment augments apoptosis in gefitinib-treated cells. Combined treatment with gefitinib and hydroxychloroquine delays tumor growth more effectively than either treatment alone. Hence, gefitinib induces autophagy as an early cellular response, and blocking autophagy may enhance the anticancer effects of EGFR-targeted therapy [343]. In wild-type and drug-resistant bladder cancer cell lines, Dasatinib treatment significantly reduces cell proliferation through G1 stage arrest. Dasatinib triggers cell death through activation of caspases, cleavage of PARP, and increased release of cytochrome c. Interestingly, it simultaneously also induces autophagy by elevating LC3-II and ATG5 expressions and decreasing p62 levels. Dasatinib suppresses the PI3K/Akt/mTOR signaling pathway by reducing the phosphorylation levels of PI3K, Akt, and mTOR, suggesting that it inhibits tumor growth by simultaneously promoting autophagy and apoptosis [344].

These findings show that there are opportunities for therapeutic intervention through the use of dual autophagy modulation strategies, like as the use of mTOR inhibitors, such as Temsirolimus, in combination with Hydroxychloroquine to create increased cytotoxicity and apoptosis in renal cell carcinoma models through decreased Phospho-S6 levels and more effective suppression of pro-survival nutrient-sensing pathways [345,346,347,348]. A list of FDA-approved drugs targeting the autophagy–apoptosis crosstalk is presented in Table 6.

## 5. Conclusions

In a biological context, the delicate interplay between autophagy and apoptosis is critical for maintaining cellular equilibrium and determining cell fate, especially in cancer. Therefore, the purpose of this review article is to describe the molecular mechanisms and signal transduction pathways involved in this delicate interaction, which may be important for developing new cancer treatments. Specifically, we reviewed how several regulatory proteins contribute to the decision-making process regarding whether a cell survives via autophagy or undergoes programmed cell death via apoptosis.

We reviewed several types of proteins that play key roles in regulating the balance between survival and cell death. These included several types of kinases (e.g., DAPK, mTOR, AMPK, p38 MAPK, MEK/ERK, JNK) and caspases, as well as heat shock proteins (HSP70, HSP90) and transcription factors (CHOP, ATF4, FOXO, HIF1α, NRF2, P53, STAT3, NF-κB). Furthermore, we described significant signal transduction pathways that enable cells to respond to external and internal signals to control this interaction, including the PI3K/Akt/mTOR pathway, the MAPK pathway, the unfolded protein response, oxidative stress, and calcium signaling. When these pathways are disrupted, cancer cells may exhibit uncontrolled growth and become resistant to therapy. UPR acts as an adaptive response when cells are exposed to mild ER stress; however, prolonged stress leads to apoptosis. Similarly, oxidative stress can have conflicting effects depending on ROS levels. Low levels of ROS promote proliferation, whereas higher levels lead to apoptosis and autophagy. Calcium signaling acts as a rheostat, allowing cells to choose between autophagic survival and apoptotic commitment in response to environmental stress. Understanding this complex relationship will open new opportunities for cancer treatment.

We discussed how both naturally occurring compounds and FDA-approved drugs are being used to manipulate autophagy–apoptosis crosstalk. Either naturally occurring compounds or FDA-approved drugs may suppress autophagy, which promotes tumor cell survival, and enhance the killing of those cells, alone or in combination with other treatments, to counteract drug resistance and improve the effectiveness of anticancer treatments. The balance between autophagy and apoptosis is delicate, providing a “fine-tuned” system that determines cell fate in the stressful tumor microenvironment. Understanding the molecular events and interactions between signaling pathways that determine this balance will provide the knowledge necessary to predict cellular outcomes. Furthermore, this knowledge will be useful for developing new, potentially more effective cancer therapies that selectively target and kill tumor cells by strategically manipulating these conserved catabolic processes.

## Figures and Tables

**Table 1:** List of Kinases and their role in autophagy–apoptosis crosstalk in cancer.

Kinase	Autophagy Targets	Apoptosis Targets	Cellular Outcome
DAPK	Beclin-1 (Thr119), Bcl-2/Bcl-XL dissociation, Vps34 complex, mTORC1 (via TSC2), cytoskeletal remodeling	p53 (Ser20), ARF, Ca^2+^ influx (GluN2B), myosin light chain	Context-dependent autophagy or apoptosis; stress adaptation and cell death
mTOR (mTORC1/mTORC2)	ULK1/2, Atg13 (by mTORC1); indirect AKT–FOXO regulation (by mTORC2)	Akt, SGK1, PKC activation	mTORC1 suppresses autophagy; mTORC2 supports survival and modulates autophagy
AMPK	ULK1, Beclin-1, TSC2, Raptor (mTORC1 inhibition)	p53; Bax; Bcl-2; Caspase-9; Caspase-3; PARP (indirectly regulated via AMPK activation and Akt–mTOR inhibition)	Induction of intrinsic (mitochondrial) apoptosis by increasing Bax/Bcl-2 ratio, activation of caspase cascade (caspase-3 via caspase 9), PARP cleavage, and suppression of cell survival signaling (Akt–mTOR), leading to programmed cell death
PI3K/Akt	mTORC1 activation, TSC1/2 inhibition	Inhibition of apoptotic pathways	Promotes growth, proliferation, and survival; suppresses autophagy
p38 MAPK	mTORC1 inhibition, ULK1 activation (oxidative stress)	p53; Bax; Bcl-2; Caspase-3	p38 enhances p53 transcriptional activity, promotes Bax-mediated mitochondrial apoptosis, inhibits anti-apoptotic Bcl-2, and activates caspase-3 to execute apoptosis
MEK/ERK	ULK1 (via AMPK), ROS-mediated autophagy	ROS-mediated cell death	Regulates autophagy and stress-induced cell death in a context-dependent manner
JNK	Bcl-2 phosphorylation, Beclin-1 release, Vps34, ATG7, LC3, p62 recruitment	ROS-mediated apoptosis (sustained activation)	Balances autophagy and apoptosis under stress conditions

**Table 2:** List of caspases and their role in autophagy–apoptosis crosstalk in cancer.

Caspase	Type	Target Substrate	Molecular Mechanism/Site	Biological Outcome
Caspase-2	Initiator	Autophagy regulatory pathways (AMPK, mTOR, MAPK)	Modulates stress-responsive signaling pathways	Suppresses basal autophagy; promotes apoptosis under cellular stress
Caspase-3	Executioner	Beclin-1	Cleavage generates a C-terminal fragment that translocate to mitochondria	Inhibits autophagy (disrupts Beclin-1–Vps34 complex); promotes apoptosis via MOMP and cytochrome c release
Caspase-3	Executioner	ATG4D	Proteolytic cleavage	Generates pro-apoptotic mitochondrial fragment
Caspase-3/-6/-8	Executioner/Initiator	ATG3	Proteolytic cleavage	Disrupts LC3 conjugation system; inhibits autophagosome formation
Caspase-3/-6/-8	Executioner/Initiator	ATG7	Proteolytic cleavage	Impairs LC3 lipidation and autophagy progression
Caspase-6	Executioner	p62 (SQSTM1)	Cleavage affecting cargo-binding function	Impairs selective autophagy and cargo recognition
Caspase-8	Initiator	ATG5, ATG12, ATG16L1	Sequestration at autophagosomal membranes limits caspase-8 activation	Autophagy suppresses apoptosis by inhibiting caspase-8 activation
Caspase-10	Initiator	BCLAF1	Downregulation via proteolytic regulation	Limits excessive autophagy; maintains survival balance

**Table 3:** List of inhibitors targeting HSP90.

Class	Specific Agent	Structural Origin/Basis	Clinical Status/Application	References
Ansamycins (1st Gen)	Geldanamycin (GA)	Benzoquinone ansamycin (natural product)	Discontinued (Hepatotoxicity)	[97,98,99,100,101]
Ansamycin Analogs	Tanespimycin (17-AAG)	Semi-synthetic (allyl-amino derivative)	Phase II/III (Hematologic/Breast)	[97,98,99,100,101]
Ansamycin Analogs	Alvespimycin (17-DMAG)	Semi-synthetic (water-soluble)	Phase I/II (Solid tumors)	[97,98,99,100,101]
Purine Analogs	BIIB021	Purine scaffold ATP mimic	Phase II (Solid tumors)	[102,103,104,105]
Benzamides	Pimitespib (TAS-116)	Selective N-terminal inhibitor	Approved in Japan for gastric cancer	[102,103,104,105]
Resorcinols (2nd Gen)	Ganetespib (STA-9090)	Triazolone/Resorcinol derivative	Failed Phase III (NSCLC)	[106,107]
Resorcinols	Luminespib (AUY922)	Isoxazole/Resorcinol derivative	Phase II (NSCLC/Breast)	[106,107]

**Table 4:** List of transcription factors modulating autophagy–apoptosis crosstalk.

Transcription Factor	Regulatory Pathway	Autophagy Targets	Apoptosis Targets	Cellular Outcome
ATF4	PERK/eIF2-a (UPR)	ATG5, ATG12, BECN1, MAP1LC3B	DDIT3 (CHOP), ASNS	Cytoprotection during acute stress; shifts to death if stress is chronic
CHOP	ATF4/ATF6 (UPR)	ATG5, ATG7, BECN1 (via BCL2 inhibition), TRB3, GADD34	BCL2 (repression), BAX, PUMA, NOXA, BIM, ERO1A	Primarily pro-apoptotic; induces cell death under severe ER stress
FOXO (1,3,4)	PI3K/Akt/mTOR, AMPK	ATG5, MAP1LC3B, BECN1	BIM	Promotes survival through nutrient recycling or apoptosis under severe deprivation
HIF1-a	Hypoxia (VHL/PHD)	BNIP3, BNIP3L	BCL2 (stabilization), BAX (context-dependent)	Mitophagy-mediated survival in hypoxic cores; metabolic reprogramming
NRF2	p62-KEAP1-NRF2	SQSTM1 (p62), autophagy-related genes	BCL2, GPX4	Antioxidant defense and survival; contributes to chemoresistance
P53	DNA Damage, ROS	DRAM, ATGs, SESN1/2	BAX, PUMA, NOXA, BCL2 (repression)	Tumor suppression; dual role in autophagy based on localization
STAT3	IL-6/JAK, Growth Factors	HIF1A, BNIP3, BECN1	BCL2, MCL1, BCL-XL	Oncogenic; cytoplasmic pool inhibits autophagy via PKR
NF-KB	IKK Complex, UPR	BECN1, SQSTM1 (p62)	BCL2, XIAP, BFL1/A1	Pro-survival and inflammatory; suppresses apoptosis; regulates p62

**Table 5:** Natural products targeting Autophagy–Apoptosis crosstalk markers.

Compound	Molecular Target	Effect on Autophagy	Effect on Apoptosis	Cancer Type	Mechanistic Crosstalk	Structure
Curcumin, Berberine	AMPK, PI3K/Akt/mTOR	Induces autophagy	Induces apoptosis	Non-small cell lung carcinoma	Activates AMPK and inhibits PI3K/Akt/mTOR → shifts balance toward apoptosis under metabolic stress	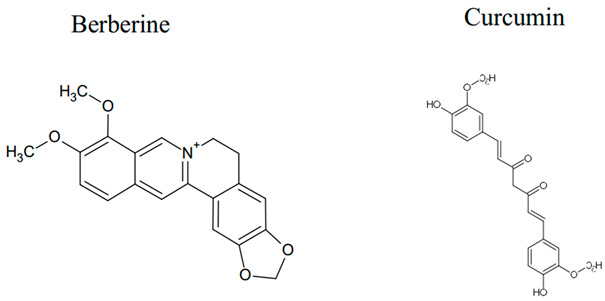
Gossypol	Bcl-2 family	Modulates autophagy	Induces apoptosis	Prostate cancer, lung cancer, breast cancer	Disrupts Bcl-2 interaction, promotes autophagy-mediated necroptosis, and apoptosis	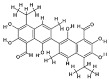
Obatoclax	Bcl-2 family	Modulates autophagy	Induces apoptosis	Leukemia, lung cancer, lymphoma	BH3 mimetic, which alters the apoptosis-autophagy balance in the cells	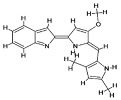
Resveratrol	Inhibits PI3K/Akt signaling	Induces Beclin-1-dependent autophagy	Activates mitochondrial apoptosis (Bax, caspase activation)	Lung cancer, breast cancer, and colon cancer	AMPK activation links autophagy induction with apoptotic signaling	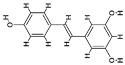
Quercetin	Ca^2+^ signaling	Modulates autophagy	Enhances apoptosis	Triple negative breast cancer, lung cancer, and colon cancer	Alters Ca^2+^ homeostasis, which enhances the apoptotic pathway in cancers	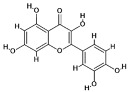
Anthocyanin	Ca^2+^ signaling	Modulates autophagy	Enhances apoptosis	Breast cancer, colon cancer	Ca^2+^ signaling is affected, which causes disruption of the survival pathways of cells	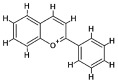
Ginsenoside K	AMPK/mTOR, JNK	Induces autophagy	Induces apoptosis	Non-small cell lung carcinoma	JNK activation with Bcl-2 disruption causes autophagy-mediated apoptosis	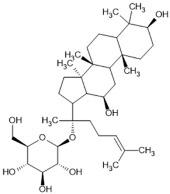
Voacamine	Beclin1	Induces autophagy-dependent cell death	No known effect on apoptosis	Osteosarcoma	Enhances doxorubicin cytotoxicity via autophagy	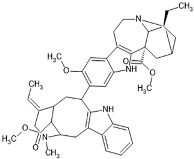
Tetrandrine	JNK, ERK	PKCα, mTOR	Induces apoptosis	Breast, Liver, Leukemia, Colon, Pancreatic cancers	JNK/ERK imbalance is caused, which in turn leads to pro-apoptotic signaling	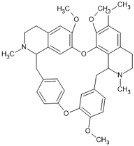
Evodiamine	JNK, Ca^2+^ signaling	Induces autophagy	Induces apoptosis	Glioblastoma	Ca^2+^ and JNK signaling are combinatorially affected, which then links autophagy with mitochondrial apoptosis	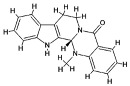
Baicalein	Inhibits PI3K/Akt/mTOR pathway	Induces autophagy through elevated LC3-II/LC3-I and Beclin-1 levels	Promotes apoptosis via mitochondrial pathway	Non-small cell lung carcinoma	PI3K/Akt inhibition coordinates autophagy activation with apoptotic cell death	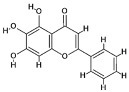
Shikonin	Inhibits PI3K/Akt/mTOR signaling.	Induces autophagy in tumor cells	Activates apoptosis and ferroptosis	Lung Cancer	Suppression of survival signaling promotes both autophagy and apoptosis	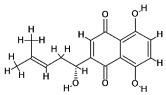
Harmine	Inhibits Akt/mTOR/p70S6K pathway	Induces autophagy	Promotes apoptosis in cancer cells	Gastric cancer	mTOR pathway suppression links autophagy induction with apoptotic signaling	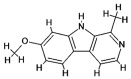
Piperlongumine	PI3K/Akt/mTOR	Inhibits autophagy	Induces apoptosis	Lung, Breast, Colon cancer	Promotes apoptosis while suppressing autophagy	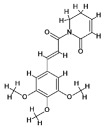
EGCG	LC3, Caspase 3	Induces autophagy	Induces apoptosis	Hepatocellular carcinoma Breast cancer	Enhances autophagic degradation of tumor proteins	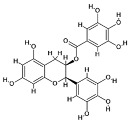
Honokiol	ROS, ERK	Induces autophagy	Induces apoptosis	Osteosarcoma, Breast cancer	ROS-mediated ERK activation takes place, and this leads to dual pathway activation	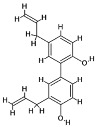
Luteolin	Mitochondrial pathway	Induces protective autophagy	Induces apoptosis	Glioblastoma, Breast cancer	Autophagy is protective; inhibition enhances apoptosis	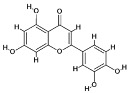
Celastrol	PI3K/Akt/mTOR STAT3	Induces autophagy	Induces apoptosis	Breast, Prostate cancer	mTOR inhibition takes place, and this leads to autophagy activation via ULK1	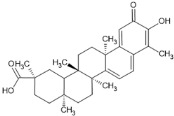
Withaferin A	PI3K/Akt/mTOR Bcl-2	Induces autophagy	Induces apoptosis	Breast cancer, Lung cancer	Inhibits Bcl-2 & Akt, which in turn promotes apoptosis and autophagy	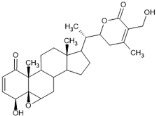

**Table 6:** FDA-approved drugs targeting Autophagy–Apoptosis crosstalk markers.

Drug	Molecular Target	Effect on Autophagy	Effect on Apoptosis	Cancer Type	Mechanistic Crosstalk	Structure
Sorafenib	Multi-kinase inhibitor	Induces cellular stress pathways, triggering autophagy and apoptosis.	Activates caspase-dependent apoptosis	Hepatocellular carcinoma. Renal cell carcinoma, differentiated thyroid carcinoma.	Autophagy is initially cytoprotective; prolonged stress shifts the balance toward apoptosis. If combined with wogonin, it can reduce autophagy and increase apoptosis.	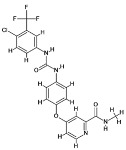
Chloroquine+ Temozolomide	EGFRvIII	Blocks the late stage of autophagy by preventing lysosomal acidification	Enhanced apoptosis due to disruption of autophagy	Glioblastoma	Autophagy blockage leads to increased accumulation of autophagosomes due to the synergy of chemotherapeutic agents; increased apoptosis	Chloroquine 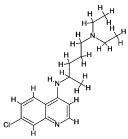 Temozolomide 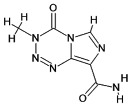
Hydroxychloroquine+ Gemcitabine+ bortezomib	Multiple molecular targets (p62, S-phase arrest and NFκB)	Disrupts autophagic flux	Enhanced apoptosis due to disruption of autophagy	Glioblastoma, pancreatic cancer, multiple myeloma	Disruption of autophagy upregulates apoptosis and sensitizes the tumor to treatment-induced stress.	Hydroxychloroquine 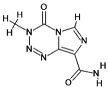 Gemcitabine 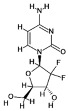 Bortezomib 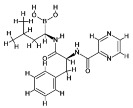
Metformin	AMPK activation; mTOR inhibition	Metformin promotes mitochondrial-dependent apoptosis in cancer cells: sensitize cancer cells to apoptosis by activating AMPK and inhibiting mTOR signaling	Enhances apoptotic signaling	Colorectal cancer, breast cancer, pancreatic, prostate cancer, lung cancer	Energy stress-induced autophagy sensitizes cells to apoptosis	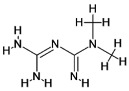
Bortezomib	Proteasome inhibition	Sensitizing cancer cells to TRAIL-mediated apoptosis.	Triggers intrinsic apoptosis	Multiple myeloma	Influence the crosstalk between autophagy and apoptosis by suppressing protective autophagy in lymphoma models and enhancing apoptosis through interactions with mTOR pathway regulators.	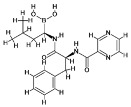
Vorinostat	Histone deacetylase	Inhibits autophagy by acetylating Beclin-1	Promotes apoptosis and cell cycle arrest	Hepatocellular carcinoma, lung cancer, breast cancer, prostate cancer, glioblastoma	Beclin-1 inhibition leads to autophagy inhibition; upregulation of apoptosis due to Bcl2 activity.	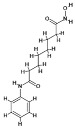
Everolimus+ Tivozanib+ Chloroquine	DR5, Caspase8 axis	Autophagy is blocked	Apoptosis is induced due to DR5/Fas-associated/Caspase8 axis	Refractory metastatic colorectal cancer	Compensatory survival apoptosis is suppressed; apoptosis is upregulated by caspase 8 activity	Everolimus 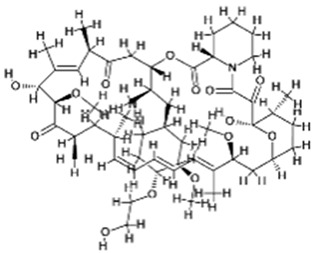 Tivozanib 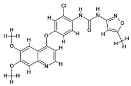
Doxorubicin	DNA, Topo II	Induces protective autophagy	Apoptosis in induced in resistant cancer cells	Breast cancer, leukemia, lymphoma	ROS and DNA damage leads to autophagy induction due to high stress; mitochondrial damage leads to apoptosis via Bax activity	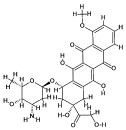
Rapamycin	mTORC1 inhibition	Strong autophagy induction	Increased tumor cell death via apoptosis	Breast Cancer	Synergy with resveratrol linking autophagy induction to apoptosis	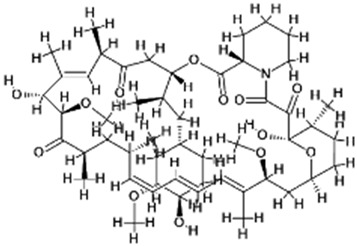
Carfilzomib	Proteasome inhibition	Induces autophagy	Activates apoptotic pathways	Myeloma	Autophagy inhibition potentiates drug-induced apoptosis (1)	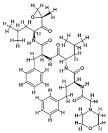
Temsirolimus + Hydroxychloroquine	mTOR inhibition + lysosomal blockade	Induction followed by autophagic flux inhibition	Enhanced apoptotic response	Melanoma	Dual modulation forces shift from protective autophagy to apoptosis (2)	Hydroxychloroquine 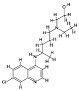 Temsirolimus 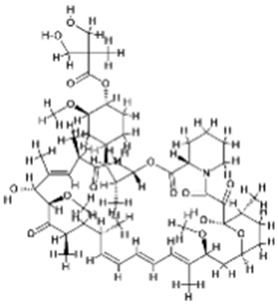
Imatinib	Tyrosine kinase receptor	Induces protective autophagy	Induces apoptosis via the formation of autophagosomes	Chronic myeloid leukemia, Glioma	The drug induces cytotoxicity and apoptosis via the formation of autophagosomes; clearance is blocked and further induces cellular stress.	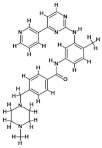
GANT61	Hedgehog signaling pathway	Induces autophagy-dependent cytotoxicity	Induces apoptosis	Hepatocellular carcinoma	Leads to drug-induced autophagy-dependent cytotoxicity, which can be reversed using other drugs like Chloroquine. Hence, making its use context dependent	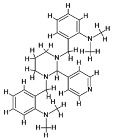
Gefitinib	EGFR; inhibits PI3K/AKT/mTOR pathway and ERK pathways	Induces autophagy (early protective response; increases autophagic flux)	Induces apoptosis (enhanced when autophagy is inhibited)	Lung cancer, Breast cancer	Gefitinib suppresses PI3K/AKT/mTOR signaling, induces autophagy and apoptosis; autophagy acts as a survival mechanism; inhibition (e.g., hydroxychloroquine) enhances apoptosis, demonstrates autophagy–apoptosis switch	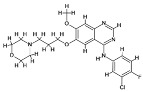
Dasatinib	SRC family kinases; inhibits PI3K/AKT/mTOR pathway	Induces autophagy (upregulates LC3-II, ATG5; downregulates p62)	Strongly induces apoptosis (caspase activation, PARP cleavage, cytochrome c release)	Bladder cancer (wild type & resistant cell lines)	Simultaneously induces autophagy and apoptosis via PI3K/AKT/mTOR suppression; autophagy and apoptosis are co-activated, with apoptosis driven by caspase signaling, coordinated crosstalk rather than purely protective autophagy	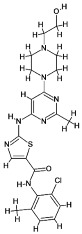

Chemical Structures obtained from PubChem and represented using ChemSketch, Version 2025 from ACDLabs.

## Data Availability

No new data were created or analyzed in this study. Data sharing is not applicable to this article.

## Abbreviations

AMPK	AMP-activated protein kinase
ARE	Antioxidant response element
ATF4	Activating transcription factor 4
ATF6	Activating transcription factor 6
ATG	Autophagy-related gene/protein
BACH1	BTB and CNC homology 1
BAX	BCL2-associated X protein
BCL2	B-cell lymphoma 2
BCLAF1	BCL2-associated transcription factor 1
BECN1	Beclin-1
BH3	BCL2 homology 3
BiP/GRP78	Binding immunoglobulin protein/Glucose-regulated protein 78
BNIP3	BCL2 interacting protein 3
BNIP3L	BCL2 interacting protein 3-like
Ca^2+^	Calcium ion
CaMKKβ	Ca^2+^/calmodulin-dependent protein kinase kinase beta
CHOP	C/EBP homologous protein
DAPK	Death-associated protein kinase
DRAM	Damage-regulated autophagy modulator
EIF2α	Eukaryotic initiation factor 2 alpha
ER	Endoplasmic reticulum
ERK	Extracellular signal-regulated kinase
FADD	Fas-associated protein with death domain
FOXO	Forkhead box O
GADD153	Growth arrest and DNA damage-inducible protein 153
GSK3β	Glycogen synthase kinase 3 beta
HIF-1α	Hypoxia-inducible factor 1 alpha
HSP	Heat shock protein
IRE1α	Inositol-requiring enzyme 1 alpha
IP3R	Inositol 1,4,5-trisphosphate receptor
JNK	c-Jun N-terminal kinase
LC3	Microtubule-associated protein 1 light chain 3
LAMP2A	Lysosome-associated membrane protein 2A
MAPK	Mitogen-activated protein kinase
MAM	Mitochondria-associated membrane
MEK	Mitogen-activated protein kinase kinase
mTOR	Mechanistic target of rapamycin
mTORC1	mTOR complex 1
mTORC2	mTOR complex 2
NF-κB	Nuclear factor kappa B
NRF2	Nuclear factor erythroid 2-related factor 2
PERK	Protein kinase RNA-like endoplasmic reticulum kinase
PHLPP	PH domain and Leucine-rich repeat Protein Phosphatase
PI3K	Phosphoinositide 3-kinase
PML	Promyelocytic leukemia protein
p38 MAPK	p38 mitogen-activated protein kinase
PP2A	Protein Phosphatase 2A
PTEN	Phosphatase and tensin homolog
PUMA	p53 Upregulated Modulator of Apoptosis
ROS	Reactive oxygen species
SQSTM1/p62	Sequestosome 1
STAT3	Signal transducer and activator of transcription 3
TSC1/2	Tuberous sclerosis complex 1/2
ULK1	Unc-51-like autophagy activating kinase 1
UPR	Unfolded protein response
VPS34	Vacuolar protein sorting 34
XBP1	X-box binding protein 1
XBP1s	Spliced X-box binding protein 1
XBP1u	Unspliced X-box binding protein 1
